# Development of a novel, sensitive translational immunoassay to detect plasma glial fibrillary acidic protein (GFAP) after murine traumatic brain injury

**DOI:** 10.1186/s13195-021-00793-9

**Published:** 2021-03-07

**Authors:** Emily B. Button, Wai Hang Cheng, Carlos Barron, Honor Cheung, Asma Bashir, Jennifer Cooper, Jasmine Gill, Sophie Stukas, David C. Baron, Jerome Robert, Elyn M. Rowe, Peter A. Cripton, Cheryl L. Wellington

**Affiliations:** 1 Djavad Mowafaghian Centre for Brain Health, University of British Columbia, 2215 Wesbrook Mall, Vancouver, British Columbia V6T 1Z3 Canada; 2 Department of Pathology and Laboratory Medicine, University of British Columbia, Vancouver, British Columbia V6T 1Z3 Canada; 3 International Collaboration on Repair Discoveries, University of British Columbia, Vancouver, British Columbia V5Z 1M9 Canada; 4 School of Biomedical Engineering, University of British Columbia, Vancouver, British Columbia V6T 1Z3 Canada

**Keywords:** GFAP, Plasma biomarker, TBI, CHIMERA, Immunoassay

## Abstract

**Background:**

Glial fibrillary acidic protein (GFAP) has emerged as a promising fluid biomarker for several neurological indications including traumatic brain injury (TBI), a leading cause of death and disability worldwide. In humans, serum or plasma GFAP levels can predict brain abnormalities including hemorrhage on computed tomography (CT) scans and magnetic resonance imaging (MRI). However, assays to quantify plasma or serum GFAP in preclinical models are not yet available.

**Methods:**

We developed and validated a novel sensitive GFAP immunoassay assay for mouse plasma on the Meso Scale Discovery immunoassay platform and validated assay performance for robustness, precision, limits of quantification, dilutional linearity, parallelism, recovery, stability, selectivity, and pre-analytical factors. To provide proof-of-concept data for this assay as a translational research tool for TBI and Alzheimer’s disease (AD), plasma GFAP was measured in mice exposed to TBI using the Closed Head Impact Model of Engineered Rotational Acceleration (CHIMERA) model and in APP/PS1 mice with normal or reduced levels of plasma high-density lipoprotein (HDL).

**Results:**

We performed a partial validation of our novel assay and found its performance by the parameters studied was similar to assays used to quantify human GFAP in clinical neurotrauma blood specimens and to assays used to measure murine GFAP in tissues. Specifically, we demonstrated an intra-assay CV of 5.0%, an inter-assay CV of 7.2%, a lower limit of detection (LLOD) of 9.0 pg/mL, a lower limit of quantification (LLOQ) of 24.8 pg/mL, an upper limit of quantification (ULOQ) of at least 16,533.9 pg/mL, dilution linearity of calibrators from 20 to 200,000 pg/mL with 90–123% recovery, dilution linearity of plasma specimens up to 32-fold with 96–112% recovery, spike recovery of 67–100%, and excellent analyte stability in specimens exposed to up to 7 freeze-thaw cycles, 168 h at 4 °C, 24 h at room temperature (RT), or 30 days at − 20 °C. We also observed elevated plasma GFAP in mice 6 h after TBI and in aged APP/PS1 mice with plasma HDL deficiency. This assay also detects GFAP in serum.

**Conclusions:**

This novel assay is a valuable translational tool that may help to provide insights into the mechanistic pathophysiology of TBI and AD.

**Supplementary Information:**

The online version contains supplementary material available at 10.1186/s13195-021-00793-9.

## Introduction

Over the past decade, impressive advances have been made in the discovery of blood biomarkers for multiple neurological indications including traumatic brain injury (TBI), Alzheimer’s disease (AD) and many others [1, 2]. Blood biomarkers have several advantages over neuroimaging and cerebrospinal fluid (CSF) biomarkers as they are less expensive, more high-throughput, and less invasive [1]. Circulating glial fibrillary acidic protein (GFAP), an intermediate filament protein expressed in astrocytes, is a particularly promising biomarker for predicting brain damage after TBI. GFAP, along with ubiquitin C-terminal hydrolase 1 (UCH-L1), was the first biomarker for concussion to be approved by the United States Food and Drug Administration (FDA) in 2018 [3] using an enzyme-linked immunosorbent assay (ELISA)-based method by Banyan Biomarkers Inc. to predict intracranial injuries on computed tomography (CT) head scan with 97.6% sensitivity. Overall, it is estimated that up to a third of TBI patients could avoid unnecessary CT scans and the associated radiation using the GFAP/UCH-L1 blood test [4]. Many other studies have supported these findings using various assays and analytical platforms [5–7] and show that post-TBI serum and plasma GFAP levels can predict magnetic resonance imaging (MRI) abnormalities [6–8], mortality [5, 9–11], delays to return to work or return to play [6, 12], and poor scores on the Glasgow Outcome Scale [5, 11, 13]. In many studies, blood GFAP was a better predictor of imaging findings or other outcomes than other biomarkers tested [5, 7, 13].

Emerging evidence suggests that circulating GFAP may also be an effective blood biomarker for other neurological indications. For example, serum GFAP correlates with multiple sclerosis (MS) disease severity [14] and tumor volume in glioblastoma patients [15]. Several studies have found an acute increase in serum or plasma GFAP after intracerebral hemorrhage [16–18] and an association between serum GFAP and stroke severity [17] or stroke volume [18]. Circulating GFAP has also shown promise as an Alzheimer’s disease (AD) biomarker in several recent studies. In a study of *n* = 28 AD patients and *n* = 34 controls, serum GFAP concentrations were higher in the AD patients and correlated with cognitive impairment [19]. In a study of *n* = 252 subjects in the Amsterdam Dementia Cohort, plasma GFAP concentrations predicted brain amyloid positivity in positron emission tomography scans and were associated with cognitive performance [20].

Despite the promise of circulating GFAP as a biomarker for multiple neurological conditions, there is currently no validated method to quantify GFAP in mouse plasma. Animal models are invaluable research tools for many neurological conditions and a robust assay of blood GFAP could catalyze many cost-effective translational studies with improved rigor by which serial blood samples could be measured. For example, murine models of TBI allow for greater control over injury severity and timing of blood collection, which can be challenging in human studies. Indeed, many animal models of TBI have been developed to better understand mechanisms of injury and potential therapeutic pathways. Our laboratory developed a closed-head TBI model called CHIMERA (Closed-Head Impact Model of Engineered Rotational Acceleration) that uses impact from a pneumatic piston resulting in rotational acceleration in mice, rats, and ferrets [21]. In a recent study using CHIMERA to induce a moderate-severe injury, mice exhibited acute neurological deficits, long-term memory deficits, neuroinflammation, axonal injury, and blood-brain barrier leakage [22].

We aimed to bridge the translational gap between human and rodent TBI research by using the Meso Scale Discovery platform to develop and validate a sensitive ELISA assay to quantify GFAP in murine plasma. We first validated assay performance according to standard operating procedures outlined by Andreasson et al. [23]. We then demonstrated that this assay reliably detects an acute and robust elevation of plasma GFAP after murine TBI, providing proof-of-concept for the utility of this assay to accelerate translational research in animal models of brain injury. We also show that plasma GFAP is elevated in aged APP/PS1 mice and in APP/PS1 mice that are deficient in plasma HDL, providing preliminary evidence supporting the use of this assay to study plasma GFAP in rodent models of AD.

## Materials and methods

### Animals and tissue collection

All procedures were approved by the University of British Columbia Committee on Animal Care (protocol A15-0096, A19-0264, A15-0040, A14-0057, A14-0003) and were performed in strict compliance with the Canadian Council on Animal Care guidelines. Animals were anesthetized with ketamine (150 mg/kg) and xylazine (20 mg/kg). A surgical plane of anesthesia was confirmed with a toe-pinch test then whole blood was collected by cardiac puncture into a tube containing ethylenediaminetetraacetic acid (EDTA). Collected blood was stored on ice up to 4 h then centrifuged to plasma at 3000*g* for 10 min at room temperature (RT) to isolate plasma. Isolated plasma was then stored at − 80 °C until analysis. To assess assay selectivity, blood collected from cardiac puncture was divided into tubes with and without EDTA to prepare plasma and serum, respectively, from the same animals. Serum was allowed to clot for 30 min at RT prior to centrifugation at 3000*g* for 10 min. Following cardiac puncture, animals were perfused with heparinized ice-cold phosphate-buffered saline (PBS). Half-brains were collected for biochemistry and snap frozen on dry ice then stored at − 80 °C. Where indicated, blood was collected from the saphenous vein immediately before anesthesia into micro capillary tubes with EDTA (14-915-85, Thermo Fisher Scientific, Waltham, MA).

### Plasma specimens for assay development and validation

Murine EDTA plasma specimens, collected as described in the “Animals and tissue collection” section, were used to create plasma pools with low, intermediate, or high expected GFAP concentrations. Expected GFAP concentrations were estimated based on observed blood GFAP concentrations in human studies. Specifically, GFAP levels have been shown to be moderately elevated in the blood of people with AD [20] and robustly elevated in blood collected within 24 h of TBI [10, 11, 13, 24–26]. Plasma specimens with low expected GFAP concentrations were collected from female wildtype C57Bl/6 mice aged 3 to 6 months (Jackson labs, line 85, mixed C57Bl/6 and C3H/HeJ backgrounds) and pooled where indicated. Plasma specimens with intermediate expected GFAP concentrations were collected from APP/PS1 mice aged 9 to 24 months and wildtype littermates aged 18 to 24 months (Jackson labs, line 85, mixed C57Bl/6 and C3H/HeJ background) and pooled where indicated. Plasma specimens with high expected GFAP concentrations were collected from mice 6 h post-TBI as described in the “Proof-of-concept studies in TBI and AD mice” section and pooled where indicated. Aliquots were stored at − 80 °C until analysis and each assay used a freshly thawed aliquot. To assess assay selectivity, human *APOE3* targeted replacement mice [27] were used in addition to C57Bl/6 mice. Targeted replacement mice were obtained from the Cure Alzheimer Fund and have humanized *APOE* introns between exons 2–3 and exons 3–4 but retain the murine ApoE 5′ and 4′ untranslated regions.

### Assay procedure

A novel highly sensitive ELISA to detect murine GFAP in plasma was developed using the Meso Scale Discovery (MSD) platform and materials detailed in Table 1. A capture and detection antibody pair reactive against mice, rats, and humans was obtained (ab244094, Abcam, Cambridge UK). This antibody pair was chosen for several reasons. First, this antibody pair was previously reported by the supplier to have cross-reactivity with mice, rats, and humans. Second, this antibody pair was available in a buffer free of sodium azide and carrier proteins, which was necessary for optimal antibody conjugation with capture and detection tags. Finally, this antibody pair was available for purchase in relatively large quantities. Biotinylation of the capture antibody was performed at a 1:10 M ratio of IgG:biotin by incubating the antibody with EZ-Link^Tm^ NHS-biotin at 4 °C overnight (20217, Sigma, Burlington MA). Labeling of the detection antibody with MSD gold sulfo-tag NHS-ester (150 nM, R91A0, Meso Scale Discovery, Rockville MD) was performed according to the manufacturer’s recommendations. Optimal capture and detection antibody concentrations were determined through a preliminary test of the signal to background ratio when measuring 100 pg/mL of recombinant GFAP protein. Recombinant GFAP stock (ab68428, Abcam, Cambridge, UK) was diluted to 10 μg/mL working stocks in 1% BSA in PBS.

**Table 1:** Assay materials

Material	Manufacturer	Catalog number
MSD GOLD Small Spot Streptavidin SECTOR pate	Meso Scale Discovery (Rockville MD)	L45SA
GFAP antibody pair	Abcam (Cambridge UK)	Ab244094
Capture label (EZ-link NHS-biotin)	Sigma (Burlington MA)	20217
Detection label (MSD gold sulfo-tag NHS-ester)	Meso Scale Discovery (Rockville MD)	R91A0 (150 nM)
Recombinant GFAP	Abcam (Cambridge UK)	Ab68428
Blocking buffer (5% BSA in PBS)	Sigma (BSA) (Burlington MA)	A7906
Assay diluent (1% BSA in PBS)	Sigma (BSA) (Burlington MA)	A7906
Wash (PBS-T)	VWR (Tween) (Radnor PA)	CAJTX251-7
Read buffer (MSD GOLD Read buffer A)	Meso Scale Discovery (Rockville MD)	R92TG
MESO QuickPlex SQ 120	Meso Scale Disocvery (Rockville MD)	AI0AA-0

Details of the assay standard operating procedure (SOP) are as follows:

1. Add blocking buffer: add 150 μL/well
  Incubate for 1 h at room temperature with shaking (150 rpm)
2. Wash: add 300 μL/well wash buffer
  Repeat 3 times, decant the wash buffer in between additions, remove excess wash buffer from the wells following the final wash by tapping on a paper towel
3. Add capture antibody: add 30 μL/well capture antibody diluted to 0.5 μg/mL in PBS
  Incubate for 1 h at room temperature with shaking (150 rpm)
4. Prepare standard curve
  Dilute recombinant protein stock first to 100,000 pg/mL in assay diluent
  Dilute from 100,000 to 40,000 pg/mL in assay diluent for the highest standard point (point 1)
  Perform 1:4 serial dilutions in assay diluent for points 2–7
  Point 8 will be blank assay diluent
5. Add samples and standards: final volume of 50 μL/well
  Standards: add 25 μL/well assay diluent plus 25 μL/well prepared standard points 1–8
  Samples: add 37.5 μL/well assay diluent plus 12.5 μL/well plasma samples
  Incubate 2 h at room temperature with shaking (150 rpm)
6. Wash: as in step 2
7. Add detection antibody: add 25 μL/well detection antibody diluted to 0.5 μg/mL in assay diluent
  Incubate for 1 h at room temperature with shaking (150 rpm)
8. Wash: as in step 2
9. Read plate: add 150 μL/well Read buffer, read plate using an MSD instrument

### Assay validation

#### Summary of assay validation parameters

A partial validation of assay performance was performed following the SOP outlined by Andreasson et al. for the BIOMARKAPD project, supported by the European Union initiative joint program for neurodegenerative disease research [23]. Validation parameters are summarized in Table 2 and detailed in the "Assay validation" sub-sections below.

**Table 2:** Parameters for partial assay validation

Parameter	Definition	Experimental details	Sample type	Technical replicates	Deviation from Andreasson et al
**Robustness**	The ability of the assay to accurately and precisely measure the analyte under small protocol variations [23].	Modify the incubation duration of capture antibody (1 h vs. 2 h vs. overnight), sample (2 h vs. 1 h vs. overnight), and detection antibody (1 h vs. 30 min) parallel assays. Analyze %CV and %recovery.	Plasma pools (low GFAP: 3–6-month-old wildtype mice, intermediate GFAP: 9–24-month-old APP/PS1 mice and 18–24-month-old wildtype littermates, high GFAP: 6 h post-TBI).	2	No deviations.
**Precision**	The variation in analyte measurement within a single assay (repeatability, intra-assay variation) and between independent assays performed on different days (intermediate precision, inter-assay variation) [23].	Repeat the assay with the same specimens on 5 independent days. Analyze intra-assay and inter-assay %CV.	Plasma pools (low GFAP: 3–6-month-old wildtype mice, intermediate GFAP: 9–24-month-old APP/PS1 mice and 18–24-month-old wildtype littermates, high GFAP: 6 h post-TBI).	5	No deviations.
**Limits of quantification**	The lowest and highest analyte concentrations in plasma that can be measured by the assay with a given level of precision (< 20% CV) [23].	LLOD: Assay replicates of diluent, calculate LLOD as 2.5 SD above the blank.	Diluent (1% BSA in PBS).	16	No recommendation available in Andreasson et al [23], followed Meso Scale Discovery recommendations.
		LLOQ: Assay replicates of diluent, calculate LLOQ as 10 SD above the blank.	Diluent (1% BSA in PBS)	16	No deviations.
		LLOQ: Assay specimens with very low GFAP concentrations. LLOQ = lowest measured concentration with duplicates < 20%CV.	Plasma specimens with low expected concentrations (*n* = 8, 3–6-month-old wildtype mice).	2	No deviations.
		ULOQ: Assay specimens with high GFAP concentrations. ULOQ = highest measured concentration with duplicates < 20%CV.	Plasma specimens with high expected concentrations (*n* = 8, 6 h post-TBI).	2	No deviations.
**Dilution linearity**	The ability of the assay to accurately and reliably detect the analyte in plasma spiked with the calibrator at a very high concentration after dilution [23]. The ability of endogenous analyte in plasma to be detected at various dilutions accurately and reliably [28].	Spike plasma specimens with recombinant protein 120-fold above the estimated ULOQ (2,000,000 pg/mL), perform serial dilutions until theoretical concentration is below LLOQ (1-fold to 1,000,000-fold). Analyze %CV and %recovery.	Plasma specimens with low expected concentrations (*n* = 3, 3–6-month-old wildtype mice).	2	No deviations.
**Parallelism**	Comparison of the signal vs. dilution factor response of the calibrator and endogenous analyte in plasma [23].	Perform serial dilutions (1-fold to 64-fold) of specimens with high expected concentration. Analyze %CV and %recovery.	Plasma specimens with high expected concentrations (*n* = 3, 6 h post-TBI).	2	3 specimens were used rather than the recommended 4 specimens, no other deviations.
		Perform serial dilutions (1-fold to 64-fold) of specimens with high expected concentration, analyze response of signal to dilution factor compared to response of recombinant protein.			Definition from Sweeney et al [28]*.* 3 specimens were used rather than the recommended 5 specimens, no other deviations.
**Recovery**	Ability to accurately and reliably measure the concentration of plasma spiked with calibrator [23].	Divide specimens into 4 aliquots, spike 3 of the aliquots with recombinant protein at concentrations across the range of the standard curve (100, 1000, and 10,000 pg/mL), add an equivalent volume of diluent to the fourth aliquot. Analyze %CV and %recovery.	Plasma specimens with low (*n* = 2, 3–6-month-old wildtype mice) and high (*n* = 3, 6 h post-TBI) concentrations.	1	No deviations.
**Stability**	The stability of the analyte in plasma after a given number of freeze-thaw cycles or a given amount of time at a given temperature [23].	Divide specimens into 19 aliquots. Aliquot 1 store at − 80 °C. Aliquots 2–6 exposed to 1, 2, 3, 5, or 7 freeze-thaw cycles (2 h at RT then store at − 80 °C), respectively. Aliquots 7–12 store at RT for 1, 2, 4, 24, 72, and 168 h, respectively. Aliquots 13–18 store at 4 °C as above. Aliquot 19 store at −20 °C for 1 month. Assay together and analyze %CV and %recovery.	Plasma pools (low: 3–6 month- old wildtype mice, intermediate: 9–24-month-old APP/PS1 mice and 18–24-month-old wildtype littermates, high: 6 h post-TBI).	2	No deviations.
**Selectivity**	The ability to measure the analyte in the presence of other substances expected to be present [23].	A) Compare assay performance in plasma and serum. B) Measure the analyte in specimens with hemolysis.	A) Plasma and serum from sham (*n* = 6) or TBI (*n* = 7) mice collected 6 h post-TBI. B) 5%, 25%, or 50% red blood cells spiked into plasma from sham (*n* = 3) or TBI (*n* = 3) mice collected 6 h post-TBI. Spiked specimens were frozen at − 80 °C then thawed and assayed.	2	Andreasson et al recommends spiking in substances with similar physiochemical structure.
**Pre-analytical factors**	The stability of the analyte with respect to blood collection and processing methods.	Blood was collected by saphenous vein and cardiac puncture. Blood from cardiac puncture was divided into 2 aliquots. One aliquot was centrifuged to plasma < 1 h after collection and one aliquot was incubated on ice 4 h before centrifugation.	Plasma from sham (*n* = 3) or TBI (*n* = 3) mice collected 6 h post-TBI.	2	No recommendation available in Andreasson et al [23]*.*

*LLOD* lower limit of detection, *LLOQ* lower limit of quantification, *ULOQ* upper limit of quantification, *TBI* traumatic brain injury, *CV* coefficient of variation, *SD* standard deviation, *RT* room temperature

#### Robustness

Assay robustness was evaluated over 6 assays run in parallel with planned variations in the duration of capture antibody, sample/standard, or detection antibody incubation. Assay 1 followed the SOP described in the “Assay procedure” section, assay 2 reduced the capture antibody incubation to 30 min, assay 3 extended the capture antibody incubation to overnight at 4 °C, assay 4 reduced the sample/standard incubation to 1 h, assay 5 extended the sample/standard incubation to overnight at 4 °C, and assay 6 reduced the detection antibody incubation to 30 min. Each assay included duplicate specimens from the low, intermediate, and high concentration plasma pools.

#### Precision

Assay precision was evaluated over 5 assays run on separate days each measuring 5 replicates from low, intermediate, and high concentration plasma pools.

#### Limits of quantification

Assay limits of quantification were determined using 2 methods. First, 8 low-concentration and 8 high-concentration specimens were assayed in duplicate to determine the lowest (lower limit of quantification (LLOQ)) and highest (upper limit of quantification (ULOQ)) concentration that could be quantified with < 20% coefficient of variation (CV). Second, 16 replicates of blank assay diluent were assayed and a calculation was made based on the standard deviation (SD) of the electrochemiluminescent (ECL) signal of the blank replicates. Lower limit of detection (LLOD) was calculated as 2.5 SD above the blank signal and LLOQ as 10 SD above the blank signal.

#### Dilution linearity

Dilution linearity of the recombinant GFAP calibrator and endogenous GFAP in plasma specimens was determined. Calibrator linearity was determined by gathering 3 plasma specimens with very low endogenous GFAP concentrations then spiking recombinant GFAP into them at a concentration 50-fold higher than the highest standard curve point. Serial dilutions of the spiked specimens were performed such that the final dilution was expected to be below the LLOD of the assay, as determined by methods described in the “Limits of quantification” section. Specimens were assayed in duplicate. Dilution linearity of endogenous GFAP in plasma was determined by gathering 3 specimens with high GFAP concentrations. Serial dilutions of each specimen were made and specimens were assayed in duplicate.

#### Parallelism

The parallelism of the response of the recombinant GFAP calibrator to that of endogenous GFAP was determined using data collected from the serial dilution of specimens with high GFAP concentrations performed as described in the “Dilution linearity” section. The ECL signal was plotted against the dilution factor for each specimen and the slope of the response curve was compared to that of diluted calibrator.

#### Spike recovery

Assay spike recovery was determined by spiking low and high concentration plasma specimens with recombinant GFAP (calibrator) at 3 concentrations. First, 2 plasma specimens with low GFAP concentrations and 3 plasma specimens with high GFAP concentrations, collected as described in the “Animals and tissue collection” and “Plasma specimens for assay development and validation” sections, were thawed and divided into 4 aliquots of 13.5 μL each. Recombinant GFAP was diluted in 1% BSA in PBS to stocks of 20,000, 2000, and 200 pg/mL. The following was then added into each aliquoted plasma specimen: 1.5 μL 1% BSA in PBS into aliquot 1, 1.5 μL of 20,000 pg/mL spike into aliquot 2, 1.5 μL of 2000 pg/mL spike into aliquot 3, and 1.5 μL of 200 pg/mL spike into aliquot 4. Each aliquot was then assayed at a 2-fold dilution. The final theoretical concentrations of recombinant GFAP in each aliquot after adjusting for dilution factor are therefore as follows: 0 pg/mL in aliquot 1 (neat aliquot), 10,000 pg/mL in aliquot 2, 1000 pg/mL in aliquot 3, and 100 pg/mL in aliquot 4. The percent recovery of each spiked specimen was then calculated as recommended by Andreasson et al [23] by the formula below:

$$ \mathrm{percent}\ \mathrm{recovery}=\frac{{\mathrm{measured}\ \mathrm{concentration}}_{\mathrm{spike}\mathrm{d}\ \mathrm{specimen}}-{\mathrm{measured}\ \mathrm{concentration}}_{\mathrm{neat}\ \mathrm{specimen}}}{{\mathrm{theoretical}\ \mathrm{concentration}}_{\mathrm{spike}}}\times 100\% $$

#### Stability

##### GFAP stability to plasma temperature modulation

The stability of GFAP in plasma exposed to various temperatures and freeze-thaw cycles was determined. Sets of 19 aliquots were prepared from low, intermediate, and high GFAP concentration plasma pools and treated as outlined by Andreasson et al. [23]. Briefly, aliquot 1 was immediately frozen and stored at − 80 °C. Aliquots 2 to 6 were exposed to 1, 2, 3, 5, or 7 freeze-thaw cycles where specimens were thawed and stored at room temperature for 2 h then stored again at − 80 °C. Aliquots 7 to 12 were stored at room temperature for 1, 2, 4, 24, 72, or 168 h before freezing at − 80 °C. Aliquots 13 to 18 were stored at 4 °C for 1, 2, 4, 24, 72, or 168 h before freezing at − 80 °C. Aliquot 19 was stored at − 20 °C for 30 days before freezing at − 80 °C. On the day of the assay, all aliquots were thawed together and assayed in duplicate.

##### Selectivity

Assay selectivity was evaluated by comparing assay measurements between serum and plasma from the same animal and investigating assay performance in the presence of hemolysis. Male and female C57Bl/6 mice, aged 2.5 to 4 months old, and *APOE3* mice, aged 5 months old, were exposed to a 2.5 J head impact with the CHIMERA device or sham procedures as described in the “Proof-of-concept studies in TBI and AD mice” section. Blood from each animal was collected 6 h after TBI by cardiac puncture, as described in the “Animals and tissue collection” section, into tubes with or without EDTA, and then processed into plasma or serum, respectively, as described in the “Animals and tissue collection” section. Plasma and serum were frozen and stored at − 80 °C then assayed together in duplicate. Thawed plasma specimens were also used to test the effect of hemolysis on assay performance. Red blood cells were pooled from the blood collected into EDTA-containing tubes from 3 sham animals. Plasma specimens were spiked with pooled red blood cells at 0%, 5%, 25%, or 50% volume. Assay diluent was added to the specimens spiked at 0%, 5%, and 25% such that an equal total volume of spike and diluent was added to each specimen. Spiked and neat specimens were frozen at − 80 °C for 1 h then thawed and assayed in duplicate.

##### Pre-analytical factors

The stability of GFAP in blood collected and processed by various methods was also determined. Male C57Bl/6 mice were exposed to a 2.5 J head impact with the CHIMERA device or sham procedures as described in the “Proof-of-concept studies in TBI and AD mice” section. Blood was collected 6 h after TBI, first by the saphenous vein and then by cardiac puncture as described in the “Animals and tissue collection” section. Blood collected by saphenous vein was stored on ice then centrifuged at 3000*g* for 10 min at room temperature within 1 h of collection. Blood collected by cardiac puncture was divided into 2 equal aliquots. Aliquot 1 was centrifuged as above within 1 h of collection. Aliquot 2 was incubated on ice for 4 h then centrifuged as above. Plasma specimens were assayed in duplicate except in the case of most saphenous blood samples where volume limitations only allowed a single measurement.

### Proof-of-concept studies in TBI and AD mice

#### CHIMERA TBI procedure

All CHIMERA procedures were approved by the University of British Columbia Committee on Animal Care (protocol A15-0096, A19-0264, and A15-0040) and were performed in strict compliance with the Canadian Council on Animal Care guidelines. Male and female C57Bl/6 mice were housed with a reverse 12 h light to dark cycle and exposed to a closed-head TBI in the sagittal plane with a CHIMERA device at 3.5 to 5 months of age as previously described [22]. Briefly, animals were anesthetized with isoflurane (induction 5%, maintenance 2–4% in oxygen 0.8 L/min), administered meloxicam (1 mg/kg) and saline (10 mL/kg) via subcutaneous injection, then placed in the supine position on the CHIMERA impactor. A toe-pinch test was used to confirm a surgical plane of anesthesia, and once confirmed, the flow of isoflurane was stopped and a single 2.5 J closed-head impact was administered using a 50-g steel piston that impacted a 3D-printed polylactic acid (PLA) interface positioned below the head to prevent skull fracture as described previously [22]. Sham animals received equivalent isoflurane, meloxicam, saline, and toe-pinch exposures without an impact. Neurological impairment was assessed using the neurological severity score (NSS) 2 h post-TBI as previously described [29]. Plasma and brains were collected as described in the “Animals and tissue collection” section.

#### Studies in APP/PS1 mice

All procedures with APP/PS1 mice were approved by the University of British Columbia Committee on Animal Care (protocol A14-0003, A14-0057) and were performed in strict compliance with the Canadian Council on Animal Care guidelines. Two APP/PS1 cohorts were included in this investigation. In the first cohort, female APP/PS1 mice and wildtype littermates (Jackson labs, line 85, mixed C57Bl/6 and C3H/HeJ background) were aged to 3, 6, 9, 12, 18, or 24 months of age. Plasma specimens were collected by cardiac puncture as described in the “Animals and tissue collection” section. In the second cohort, APP/PS1 mice with normal plasma HDL cholesterol (HDL-C) or with plasma HDL-C deficiency were generated as previously described [30]. Briefly, APP/PS1 mice (Jackson laboratories, B6.Cg-Tg (APPswe,PSEN1dE9)85Dbo/Mmjax, MMRRC stock no: 34832-JAX) on a C57Bl/6 background were first bred with apolipoprotein A-I (apoA-I)-deficient mice (Jackson Laboratories, B6.129P2-*Apoa1*^*tm1Unc*^/J, Stock no: 002055, also on a C57Bl/6 background) to produce an F1 generation hemizygous for both *apoa1* and the APP/PS1 transgenes. These animals where then backcrossed to apoA-I-deficient mice to produce F2 male and female mice of four genotypes: APP/PS1 mice hemizygous for *apoa1* (APP/PS1 apoA-I^HEM^), APP/PS1 mice with complete *apoa1* deficiency (APP/PS1 apoA-I^KO^), nontransgenic littermates hemizygous for *apoa1* (WT apoA-I^HEM^), and nontransgenic littermates deficient in *apoa1* (WT apoA-I^KO^). ApoA-I is the primary protein component of plasma HDL; therefore, apoA-I^KO^ mice have drastically reduced plasma HDL-C levels, as has been previously shown [30]. Plasma specimens were collected from these mice at 12 months of age as described in the “Animals and tissue collection” section.

### Brain biochemistry

Brain homogenization and biochemistry were performed by an investigator blinded to animal injury status. Frozen brains were homogenized using a Tissuemite homogenizer in 8 volumes of ice-cold radioimmunoprecipitation (RIPA) buffer (5 mM EDTA, 50 mM NaCl, 10 mM Na Pyrophosphate, 50 mM NaF, 1% NP40 alternative, pH 7.4) containing a complete protease inhibitor (11836153001, Millipore Sigma, Burlington, MA), as previously described [22, 30]. RIPA homogenates were centrifuged at 9000 rpm for 10 min at 4 °C and the supernatants were stored at − 80 °C until analysis. Interleukin 6 (IL-6) was measured using a multiplex ELISA with samples diluted 1:2 in supplied dilution buffer (V-PLEX Proinflammatory Panel 1 Mouse Kit, K15048D, Meso Scale Discovery, Rockville MD). Analyte concentrations in the homogenate were normalized to total protein concentrations measured using a bicinchoninic acid (BCA) assay (23225, Thermo Fisher, Waltham MA).

### Statistics

Most statistical analyses were performed with GraphPad Prism 7, and *p* values < 0.05 were considered statistically significant. One-way ANOVA followed by Dunnett’s or Tukey’s multiple comparisons test were used to compare groups to a control group or to all other groups, respectively. One-way ANOVA with repeated measures followed by Dunnett’s multiple comparisons test was used to compare the effects of hemolysis on assay performance. Paired *t* tests were used to compare the effects of serum vs. plasma, route of blood collection, and time before centrifugation into plasma. Where indicated, data were log transformed before analysis. Differences in plasma GFAP in the proof-of-concept TBI and AD studies were analyzed by two-way ANOVA followed by Sidak’s or Tukey’s multiple comparisons test with TBI exposure, time, age, APP/PS1 genotype, or apoA-I genotype as factors. Coefficients of variation were calculated as standard deviation divided by mean.

Precision data were also analyzed with a mixed model with maximum likelihood using SPSS Statistics. Full model syntax and output is available in Additional File 1. Plasma group (low, intermediate, or high concentration plasma pool) was analyzed as a fixed variable, technical replicate (intra-assay variation) as a repeated variable and a random variable, and assay replicate (inter-assay variation) as a random variable. Three additional models were run with each plasma group analyzed separately and no fixed variable included.

GFAP concentrations were interpolated from an 8-point standard curve by nonlinear regression using a 4 parameter logistic (4PL) equation. Standard curve concentrations and ECL signal were log transformed and curve fitting was performed using GraphPad Prism 7.

## Results

### Assay development and analytical validation overview

To enable plasma GFAP to be quantified in preclinical specimens, we used the MSD platform to develop a novel, highly sensitive ELISA assay based on a GFAP capture-detection antibody pair from Abcam (ab244094, Abcam, Cambridge, UK). The assay requires 12.5 μL of murine plasma and can be run in 5 h. We performed a partial validation of this assay using published SOPs for immunoassay validation endorsed by BIOMARKAPD [23], a consortium of scientists aiming to develop biomarker assays for AD and Parkinson’s disease. Intra-assay CV is 5.0% and inter-assay CV is 7.2%. The assay has excellent dynamic range, with an LLOD, LLOQ, and ULOQ of 9.0, 24.8, and 16,533.9 pg/mL, respectively. The assay shows dilution linearity over a wide range of calibrator and plasma specimen dilutions. Calibrator can be diluted from 200,000 to 20 pg/mL with 90–123% recovery, and plasma specimens with high GFAP concentrations can be diluted up to 32-fold with 96–112% recovery. Spike recovery is 67–100% depending on the concentration of the spike and the endogenous GFAP concentration in the specimens with the best recovery for low-concentration spikes into high-concentration specimens and the worst for low-concentration spikes into low-concentration specimens. GFAP concentrations measured by the assay remain stable after exposure of plasma specimens to up to 7 freeze-thaw cycles, 168 h at 4 °C, 24 h at RT, or 30 days at − 20 °C. GFAP can also be detected in serum using this assay however measured concentrations in serum are on average 1.18-fold higher than measurements in plasma. Specimen hemolysis can suppress assay measurements with increased suppression observed with increased hemolysis severity. In terms of pre-analytical factors, storage of blood for up to 4 h on ice before centrifugation to plasma does not affect assay performance but measurements may be biased when specimens are collected from the saphenous vein rather than cardiac puncture. We were unable to perform validation for the trueness and uncertainty of the assay due to the lack of available certified reference material. Detailed results for these performance parameters are provided below.

### Robustness of the assay to small protocol modifications

**Fig. 1:**
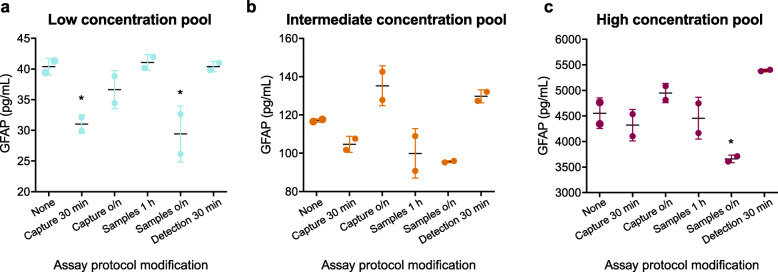
The assay is robust to changes in antibody incubation duration but less robust to changes in samples incubation duration. **a** Low-, **b** intermediate-, and **c** high-concentration plasma pools were assayed using the standard assay protocol (1 h capture antibody incubation, 2 h sample incubation, 1 h detection antibody incubation) and several protocols with modifications to incubation durations. Robustness to protocol modifications was assessed by observing the variation in sample duplicate measurements and the closeness of the measured sample concentrations under each protocol to the measurements under the standard protocol. Mean concentration of duplicates is plotted with each point representing an individual sample replicate and error bars representing ± SD. Differences in measured sample concentration compared to the standard assay protocol were analyzed by one-way ANOVA with Dunnett’s multiple comparisons test and displayed within the graphs as *p < 0.05. o/n: overnight, SD: standard deviation

Assay robustness to small protocol variations was assessed over 6 assays run in parallel following the protocol variations described in Table 3. Overall, coefficients of variation (CV) of specimen duplicates all remained < 15% with the exception of conditions where the plasma specimen incubation time was modified (Table 3). Specifically, extending specimen incubation time from 2 h to overnight resulted in a CV of 18.2% for low-concentration plasma pool duplicates. Capture antibody incubation time had little effect on duplicate variation when the time was reduced from 1 h to 30 min (< 7.5%) or when the time was extended to overnight (< 10%). Reduction of the detection antibody incubation time from 1 h to 30 min also had little effect on duplicate variation (< 2.5% CV). Variations in specimen duplicates are also illustrated in Fig. 1.

**Table 3:** Assay protocol modifications and summary of assay robustness results

Assay	Modification	Plasma pool	Replicate CV (%)	%recovery*
1	Standard protocol (Capture 1 h, Samples 2 h, Detection 1 h)	Low	3.7	–
		Intermediate	0.7	–
		High	6.7	–
2	Capture 30 min	Low	6.2	76.8
		Intermediate	3.7	89.4
		High	7.4	94.8
3	Capture overnight	Low	9.6	90.7
		Intermediate	7.1	115.5
		High	3.9	108.7
4	Samples 1 h	Low	3.4	101.7
		Intermediate	11.2	85.2
		High	9.7	97.9
5	Samples overnight	Low	18.2	72.7
		Intermediate	0.5	81.6
		High	2.0	80.4
6	Detection 30 min	Low	2.4	100.1
		Intermediate	2.3	110.8
		High	0.4	118.4

*%recovery = (concentration measured with modified assay protocol/concentration measured with standard assay protocol) × 100%. *CV* coefficient of variation

The closeness of the measured sample concentrations between different assay protocols was also evaluated by calculating the percent recovery compared to the standard protocol as detailed in Table 3 and illustrated in Fig. 1. Percent recovery ranged from 80 to 119% in all cases with two exceptions. When specimen incubation time was extended to overnight, the measured concentration of the low plasma pool was only 72.7% of that measured with the SOP. When capture antibody incubation time was reduced from 1 h to 30 min, the percent recovery of the low-concentration plasma pool was 76.8%.

Overall, all issues with assay robustness were restricted to measurements of the low-concentration plasma pool. This finding is not surprising given the greater variability in measurements of specimens with low concentrations under the standard protocol as described below in the "Repeatability and intermediate precision of the assay" section. As the measured concentrations of the low-concentration plasma pool are relatively close to the LLOQ (24 pg/mL as described in the “Assay limits of quantification and detection” section), higher variation is to be expected compared to measurements of intermediate and high-concentration plasma pools, which have concentrations closer to the middle of the quantification range of the assay as described in the “Assay limits of quantification and detection” section. The percent recovery of low-concentration specimens was particularly affected by two protocol modifications: reducing the capture antibody incubation duration (protocol 2) and extending the specimen incubation duration (protocol 5).

In protocol 2, the percent recovery issues for low-concentration specimens may be due to incomplete binding of the capture antibody to the plate. We suspect that capture antibody binding to the plate may be incomplete by 30 min but reaches saturation by 1 h. In protocol 5, the issues with variation and percent recovery for low-concentration specimens may be due to differences in standard curve preparation. The standard curves for protocols 1–4 and protocol 6 were prepared simultaneously. However, the standard curve for protocol 5 was prepared separately on the day before the other protocols to allow for overnight specimen incubation. As measurements at the low end of the standard curve are especially sensitive to changes in standard curve preparation, this may explain why variation and percent recovery of the low-concentration pool was relatively poor in this case.

### Repeatability and intermediate precision of the assay

**Fig. 2:**
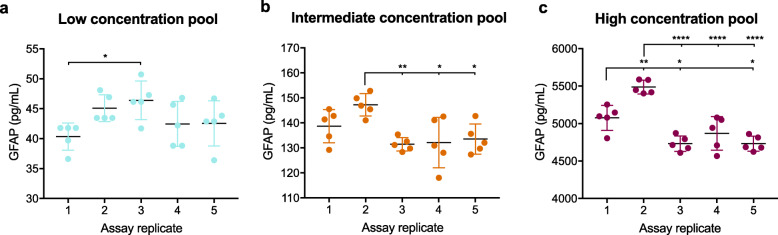
High intra-assay and inter-assay precision is observed for low-, intermediate-, and high-concentration samples. Assay precision was determined through the measurement of **a** low-, **b** intermediate-, and **c** high-concentration plasma pools over 5 days with 5 sample replicates per day. Mean concentration of replicates is plotted with each point representing an individual sample replicate and error bars representing ± SD. Differences in measured sample concentration between assay replicates were analyzed by one-way ANOVA with Tukey’s multiple comparisons test and displayed within the graph as *p < 0.05, **p < 0.01, and ****p < 0.0001. SD: standard deviation

Specimens from low-, intermediate-, and high-concentration plasma pools were repeatedly assayed over 5 separate days with 5 replicates each day to evaluate intermediate precision and repeatability of the assay, respectively (Fig. 2). Although mean concentrations were significantly different between some assay replicates, CV for repeatability and intermediate precision were all < 10% as detailed in Table 4. Overall, specimens from the low-concentration plasma pool showed the lowest repeatability and intermediate precision whereas specimens from the high-concentration pool showed the best repeatability and intermediate precision. Mean repeatability (intra-assay variation) across all pools was 5.0% and mean intermediate precision (inter-assay variation) was 7.2%.

**Table 4:** Assay repeatability and intermediate precision

Plasma pool	Mean concentration (pg/mL)	Repeatability CV (%) (intra-assay) (***n*** = 5)	Intermediate precision CV (%) (inter-assay) (***n*** = 5)
Low	43.4	7.2	8.5
Intermediate	136.6	4.7	6.5
High	4979.9	2.9	6.9
**Mean**	1720.0	**5.0**	**7.2**

*CV* coefficient of variation

Precision data were also analyzed with a mixed model whereby plasma pool (low, intermediate, or high GFAP concentration pool) was analyzed as a fixed variable, assay date (inter-assay variation) was analyzed as a random variable, and technical replicate (intra-assay variation) was analyzed as a nested random variable within assay date. Detailed results are available in Additional File 1. We found that the greatest source of error was the plasma pool, with a standard error of 93.5 pg/mL. Inter-assay and intra-assay variations had standard errors of 59.6 pg/mL and 26.4 pg/mL, respectively. Coefficients of variation calculated from variance data produced by the mixed model were similar to the CVs reported in Table 4 with a total intra-assay CV of 3.4% and inter-assay CV of 7.7% (Table 5).

**Table 5:** Mixed model analysis estimates of fixed effects and covariance

Plasma pool	Mean concentration (pg/mL)	Group variation	Intra-assay variation				Inter-assay variation			
		SE (pg/mL)	Variance (pg/mL)	SD (pg/mL)	SE (pg/mL)	%CV	Variance (pg/mL)	SD (pg/mL)	SE (pg/mL)	%CV
**All (model 1**^**A**^**)**	1720.0	93.5	3484.7	59.0	26.4	3.4	17,763.2	133.3	59.6	7.7
**Low (model 2**^**B**^**)**	43.4	–	4.0	2.0	0.9	4.6	3.5	1.9	0.8	4.3
**Intermediate (model 3**^**C**^**)**	136.6	–	23.5	4.8	2.2	3.5	24.2	4.9	2.2	3.6
**High (model 4**^**D**^**)**	4979.9	–	0.0	0.0	0.0	0.0	70,844.3	266.2	119.0	5.3

^A^All groups: plasma pool = fixed effect, technical replicate = random effect nested in assay date, assay date = random effect. ^B^Low plasma pool: technical replicate = random effect nested in assay date, assay date = random effect. ^C^Intermediate plasma pool: technical replicate = random effect nested in assay date, assay date = random effect. ^D^High plasma pool: technical replicate = random effect nested in assay date, assay date = random effect. SD for intra-assay and inter-assay variation were calculated as follows: SD= √variance). SE for intra-assay and inter-assay variation were calculated as follows: SE = SD/(√*n*), *n* = 5. *SE* standard error, *SD* standard deviation, *CV* coefficient of variation

### Assay limits of quantification and detection

**Fig. 3:**
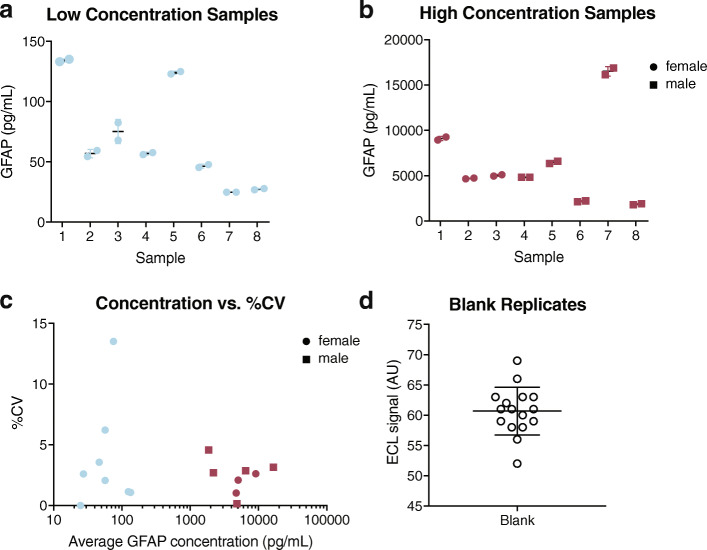
The assay has a wide quantitative dynamic range from 25 pg/mL to at least 16,533 pg/mL and a lower limit of detection of 9 pg/mL. Eight plasma samples with **a** very low or **b** very high expected GFAP concentrations were assayed in duplicate and **c** CV of duplicates were determined. **d** 16 blank replicates (1% BSA in PBS) were assayed to calculate an estimated lower limit of quantification and lower limit of detection as described in Table 6. **a**,**b**,**d** Mean concentration of replicates is plotted with each point representing an individual sample replicate and error bars representing ± SD. **c** Points represent mean sample concentration and CV of duplicates. **a**–**c** Circles represent samples from female mice, squares represent samples from male mice. AU: arbitrary units, CV: coefficient of variation, SD: standard deviation, BSA: bovine serum albumin, PBS: phosphate-buffered saline, ECL: electrochemiluminescence

Two methods were used to establish assay limits of quantification and detection. First, 8 plasma specimens with low (Fig. 3a) or high (Fig. 3b) GFAP concentrations were assayed in duplicate and the CV was evaluated (Fig. 3c). As all duplicates were within 20% CV, the actual LLOQ may be lower and the actual ULOQ may be higher than reported in Table 6. Second, LLOD and LLOQ were calculated from the variation in ECL signal from 16 blank replicates (Fig. 3d) where LLOQ is 10 SD above the blank measurement and LLOD is 2.5 SD above the blank measurement (Table 6). The calculated LLOQ (23.7 pg/mL) closely resembled the estimated LLOQ from low sample measurement (24.8 pg/mL) suggesting that the true LLOQ of the assay is approximately 24 pg/mL.

**Table 6:** Limits of quantification and detection

Measured LLOQ	Measured ULOQ	Calculated LLOQ	Calculated LLOD
24.8 pg/mL*	16,533.9 pg/mL*	23.7 pg/mL^#^	9.0 pg/mL^†^

^*^All sample replicates < 20% CV; therefore, actual LLOQ may be lower and ULOQ may be higher than reported here. ^#^calculated as 10 SD above blank measurement from 16 blank replicates. ^†^calculated as 2.5 SD above blank measurement from 16 blank replicates. *LLOQ* lower limit of quantification, *ULOQ* upper limit of quantification, *LLOD* lower limit of detection, *CV* coefficient of variation, *SD* standard deviation

### Dilution linearity of recombinant GFAP spiked into plasma and endogenous plasma GFAP

**Fig. 4:**
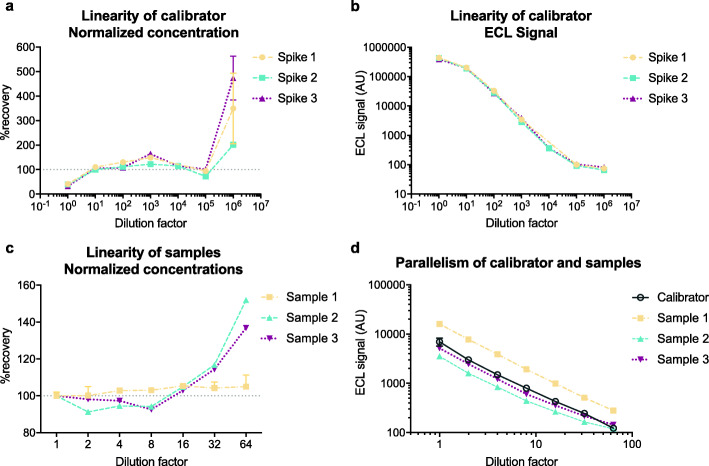
The assay shows high dilution linearity over a wide range of calibrator and plasma specimen dilutions. Assay calibrator was spiked into 3 individual plasma samples with very low endogenous GFAP to a concentration of 2,000,000 pg/mL. Serial dilutions from 10-fold to 1,000,000-fold were made and samples at each dilution step were assayed in duplicate. **a** Percent recovery of samples spiked with calibrator plotted against dilution factor. **b** ECL signal of samples spiked with calibrator were plotted against the dilution factor. Serial dilutions were performed from 2-fold to 64-fold on 3 plasma samples with high endogenous GFAP concentrations. **c** Percent recovery of diluted plasma samples plotted against dilution factor. **d** ECL signal of diluted plasma samples and calibrator plotted against dilution factor. Points represent mean concentration of duplicates and error bars represent ±SD. For some points, the error bars are shorter than the height of the symbol and therefore are not shown. AU: arbitrary units, ECL: electrochemiluminescence

Dilution linearity of recombinant GFAP calibrator in the assay was determined by spiking calibrator into plasma specimens with very low endogenous GFAP. Three specimens were spiked at a concentration 50-fold higher than the highest point in the calibration curve and 121-fold higher than the minimum ULOQ determined in the “Assay limits of quantification and detection” section (Table 6). Serial dilutions were performed to yield an expected concentration lower than the LLOD and specimens were assayed in duplicate. The percent recovery at each dilution factor was plotted (Fig. 4a) and detailed in Table 7. Duplicate variation was < 15% CV, and percent recovery was between 90 and 123% for dilution factors from 10-fold to 100,000-fold, corresponding to concentrations of 20 to 200,000 pg/mL. Undiluted specimens and specimens diluted 1,000,000-fold showed poor-mean percent recoveries of 37.6 ± 4.3% and 369.2 ± 136.2%, respectively. Dilutions in the 10-fold to 100,000-fold range also showed a linear ECL signal response (Fig. 4b). Overall, these data suggest that very high concentrations of GFAP can be diluted into the working range of the assay with acceptable percent recovery and levels of variation.

**Table 7:** Dilution linearity of plasma spiked with recombinant GFAP protein

Dilution factor	Spike concentration (pg/mL)	Mean duplicate CV (%)	%recovery* (mean ± SD) (***n*** = 3)
1:1	2,000,000	11.5	37.6 ± 4.3
1:10	200,000	5.7	105.0 ± 4.9
1:100	20,000	9.8	116.4 ± 12.1
1:1000	2000	14.9	123.1 ± 40.9
1:10,000	200	3.9	114.5 ± 0.5
1:100,000	20	13.9	90.5 ± 15.0
1:1,000,000	2	38.1	369.2 ± 136.2

*%recovery = (measured concentration adjusted for dilution/theoretical concentration) × 100%

Dilution linearity of endogenous GFAP in plasma specimens was similarly evaluated by creating serial dilutions of 3 plasma specimens with high endogenous GFAP and investigating the percent recovery (Fig. 4c) and ECL signal response linearity (Fig. 4d) across a range of dilutions from 1-fold to 64-fold. Average duplicate CV was < 7% at all dilution factors (Table 8). Mean CV from neat specimen concentration was less than 20% across all dilution factors and less than 10% up to a 32-fold dilution. Mean percent recovery was between 96 and 112% up to a 32-fold dilution but grew to 131.2 ± 23.9% at a 64-fold dilution. Overall, these data suggest that plasma samples with high GFAP concentrations can be diluted up to 32-fold with excellent recovery and variation (Table 8).

**Table 8:** Dilutional linearity of endogenous GFAP in plasma samples

Dilution factor	Mean duplicate CV (%)	Mean CV from neat specimen (%)	%recovery* (mean ± SD) (***n*** = 3)
1:1	2.7	–	–
1:2	3.8	2.6	96.5 ± 4.7
1:4	1.6	2.6	98.2 ± 4.2
1:8	2.9	3.9	96.6 ± 5.6
1:16	0.8	3.0	104.4 ± 1.3
1:32	1.4	7.8	111.8 ± 6.7
1:64	6.2	18.2	131.2 ± 23.9

*%recovery = (measured concentration adjusted for dilution/measured concentration of undiluted sample) × 100%. *CV* coefficient of variation, *SD* standard deviation

Excellent parallelism between endogenous GFAP in plasma specimens and recombinant GFAP calibrator was also demonstrated by plotting the ECL signal against dilution factor and observing the slopes of the responses (Fig. 4d).

### Recovery of recombinant protein spiked into plasma at various concentrations

**Fig. 5:**
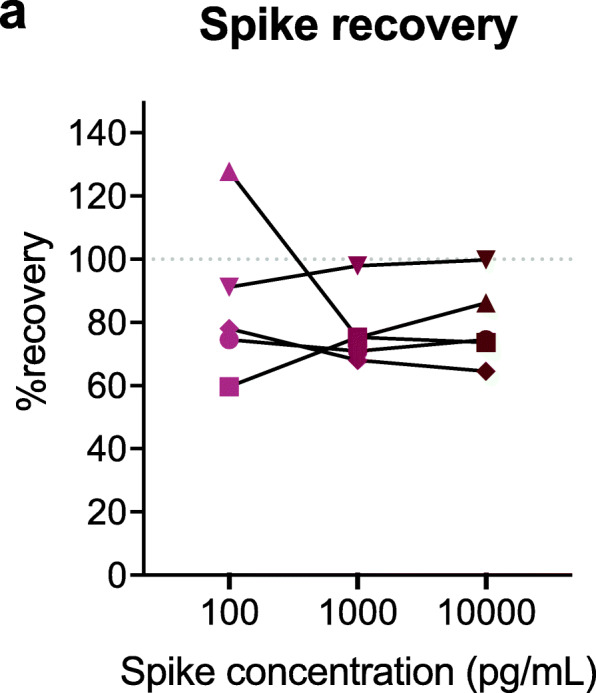
Spike recovery of the assay is optimal for specimens with high endogenous GFAP concentrations and sub-optimal for specimens with low endogenous GFAP concentrations. **a** Five plasma samples were spiked with calibrator at 0, 100, 1000, or 10,000 pg/mL. Mean percent recovery is plotted at each spike concentration with points representing individual spiked sample percent recovery and error bars representing ±SD. Point shapes represent individual plasma samples. %recovery = ((measured concentration spiked sample-measured concentration neat sample)/concentration of spike) × 100%. SD: standard deviation

The ability of the assay to accurately measure GFAP in plasma spiked with calibrator was tested using 5 plasma specimens spiked with 0, 100, 1000, or 10,000 pg/mL of calibrator (Fig. 5). The mean percent recovery overall was 81.2 ± 17.3% (Table 9). Percent recovery was better overall in spiked specimens with higher endogenous GFAP concentrations (87.7 ± 19.5%) compared to spiked specimens with lower endogenous GFAP concentrations (71.4 ± 6.05). Percent recovery was best when a low-concentration spike (100 pg/mL) was added to specimens with high endogenous GFAP concentrations (99.9 ± 25.8%) and worst when low-concentration spikes (100 pg/mL) were added to specimens with low endogenous GFAP concentrations (67.1 ± 10.5%).

**Table 9:** Percent recovery of low-concentration and high-concentration plasma spiked with recombinant GFAP

Spike concentration (pg/mL)	%recovery* (mean ± SD)		
	All samples	Low-concentration samples (***n*** = 2)	High-concentration samples (***n*** = 3)
100	86.3 ± 25.8	67.1 ± 10.5	99.0 ± 25.8
1000	77.5 ± 11.8	73.1 ± 3.1	80.4 ± 15.6
10,000	79.7 ± 13.6	74.1 ± 0.6	83.5 ± 17.8
Mean	81.2 ± 17.3	71.4 ± 6.0	87.7 ± 19.5

*%recovery = ((measured concentration spiked sample-measured concentration neat sample)/concentration of spike) × 100%. *SD* standard deviation

### Assay stability to plasma temperature variations

**Fig. 6:**
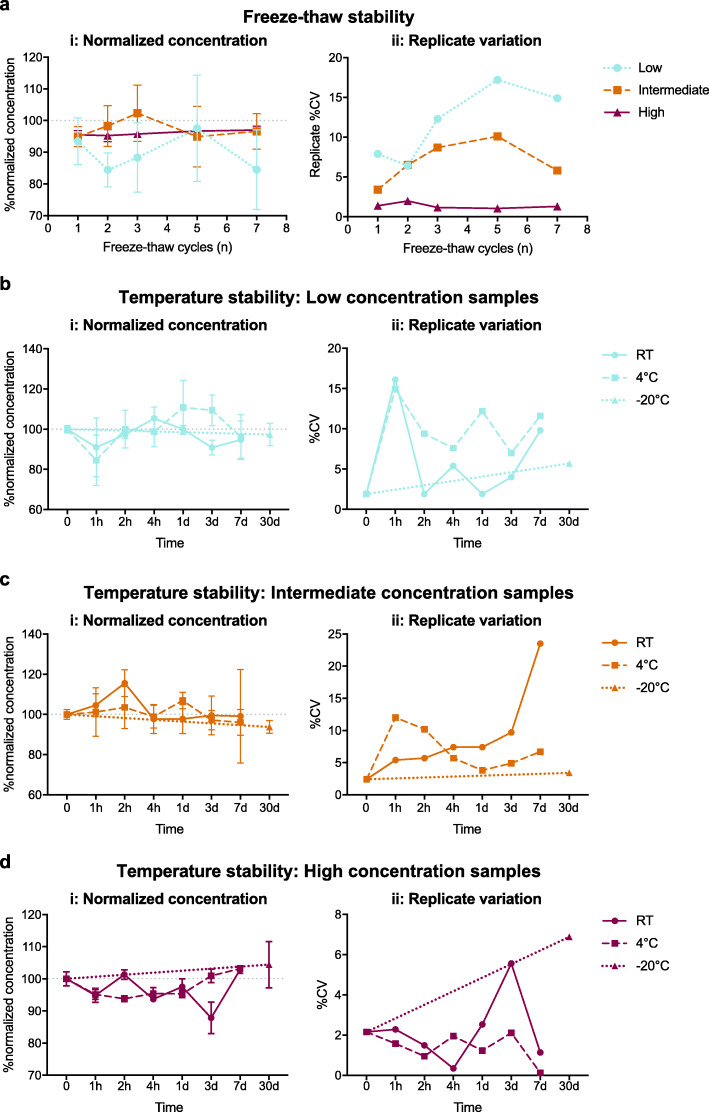
Assay measurements remain stable in specimens exposed to up to 7 freeze-thaw cycles, 168 h at 4 °C, 24 h at room temperature, or 30 days at − 20 °C. **a** Samples from low-, intermediate-, and high-concentration plasma pools were assayed after a number a freeze-thaw cycles. Samples from **b** low-, **c** intermediate-, and **d** high-concentration plasma pools were also assayed after incubation at 4 °C, room temperature, or − 20 °C. (i) Normalized concentrations relative to samples directly stored at − 80 °C and (ii) variation in sample duplicates are plotted. Points represent (i) mean normalized concentration or (ii) CV of duplicates and (i) error bars represent ±SD. For some points, the error bars are shorter than the height of the symbol and therefore are not shown. The effect of plasma temperature modification on measured concentration was analyzed by repeated measures one-way ANOVA with Dunnett’s multiple comparisons test; no significant differences were found. CV: coefficient of variation, RT: room temperature

The stability of plasma GFAP to freeze-thaw cycles and storage at room temperature, 4 °C, or − 20 °C for various lengths of time was measured for specimens from low-, intermediate-, and high-concentration pools. Overall, high-concentration pool specimens were remarkably stable. Percent recovery compared to specimens with no temperature modulation was between 95 and 97% after up to 7 freeze-thaw cycles (Fig. 6ai), between 92 and 103% after up to 168 h at 4 °C (Fig. 6di), between 93 and 96% after up to 24 h at room temperature (Fig. 6di), and between 88 and 103% after up to 168 h at room temperature (Fig. 6di). Duplicate variation for specimens from the high-concentration pool were within 4% CV across all conditions described above (Fig. 6aii, dii).

Plasma GFAP from the low-concentration pool was less stable yet still showed a percent recovery between 84 and 111% (Fig. 6ai, bi) and duplicate CV within 12% for all conditions (Fig. 6aii, bii). Plasma from the intermediate-concentration pool showed percent recoveries similar to specimens from the high-concentration pool (between 93 and 115%) (Fig. 6ai, ci); however, duplicate variation was higher. Specifically, duplicate variation was within 8% in most conditions and reached 16.6% in specimens stored at room temperature for 168 h (Fig. 6aii, cii).

Storage at − 20 °C for 30 days had little impact with percent recoveries of 97.4%, 93.7%, and 104.4% for low-, intermediate-, and high-concentration pools, respectively (Fig. 6bi, ci, di). Duplicate variations also remained low after 30 days at − 20 °C with CV of 4.1%, 2.4%, and 6.9% for low-, intermediate-, and high-concentration pools, respectively (Fig. 6bii, cii, dii).

### Assay performance in serum and in specimens with hemolysis

**Fig. 7:**
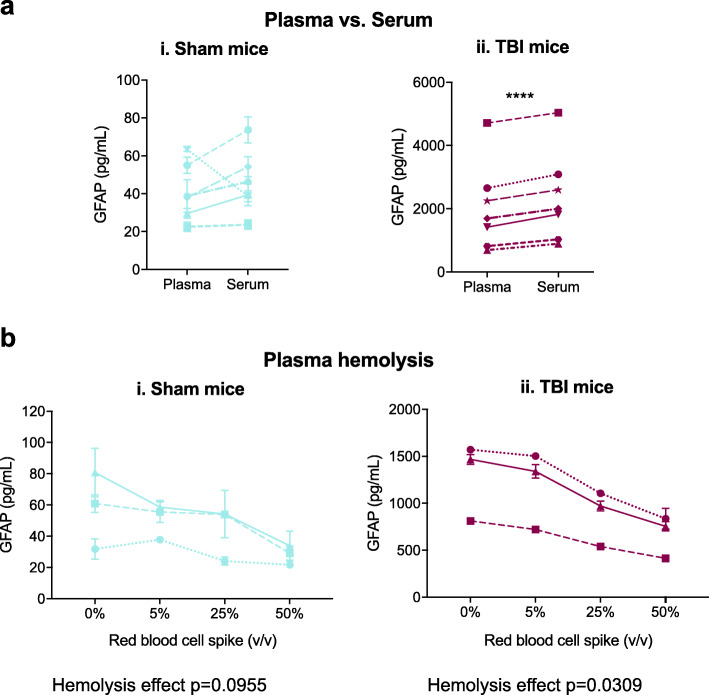
Assay measurements are slightly higher in serum than in plasma and are reduced in the presence of hemolysis. Blood was collected from mice 6 h after isoflurane exposure (sham) (i) or isoflurane exposure and TBI (ii) by cardiac puncture into tubes with (plasma) and without (serum) EDTA. **a** Plasma and serum were isolated by centrifugation and were assayed in duplicate. Differences in concentrations by matrix were analyzed by paired *t* test, ****p < 0.0001. **b** Plasma specimens were spiked with 0%, 5%, 25%, or 50% red blood cells, frozen at − 80 °C for 1 h, thawed, and assayed in duplicate. The effect of hemolysis on measured concentrations was analyzed by one-way ANOVA with repeated measures (exact *p* values are shown below graphs) followed by Dunnett’s multiple comparisons test, no significant differences from neat plasma were found. Mean plasma GFAP concentrations for each mouse are plotted with points representing mean and error bars ±SD of duplicates, in some cases error bars are smaller than the symbol. TBI: traumatic brain injury, EDTA: ethylenediaminetetraacetic acid, SD: standard deviation

The selectivity of the assay was evaluated by investigating the ability of the assay to detect GFAP in serum compared to plasma and the ability to detect GFAP in plasma specimens with hemolysis. GFAP concentrations measured in serum were slightly elevated compared to GFAP concentrations measured in plasma from the same animal. This difference was not significant in sham animals (Fig. 7ai) (1.16-fold. *p* = 0.4901 by paired t test) but was significant in TBI animals (Fig. 7aii) (1.20-fold, *p* < 0.0001 by paired *t* test). Duplicate variation was similar between plasma and serum measurements (Table 10).

**Table 10:** Duplicate variation in plasma and serum specimens

Matrix	Duplicate variation (%CV)	
	Sham (*n* = 6)	TBI (*n* = 7)
**Plasma**	9.3	11.4
**Serum**	2.7	3.6

*CV* coefficient of variation, *TBI* traumatic brain injury

The effect of hemolysis on assay performance was evaluated by spiking red blood cells into sham (Fig. 7bi) and TBI (Fig. 7bii) plasma specimens at various concentrations then freezing the spiked plasma at − 80 °C for 1 h. Hemolysis reduced measured GFAP concentrations, with a non-significant effect observed in plasma from sham animals (Fig. 7bi) (*p* = 0.0955 by repeated measures one-way ANOVA) and a significant effect in plasma from TBI animals (Fig. 7bii) (*p* = 0.0309 by repeated measures one-way ANOVA). Spiking red blood cells into plasma at 50% by volume reduced the measured concentration to 52.3% of the concentration measured in neat plasma overall. Spiking red blood cells into plasma at 5% by volume only reduced the measured concentration to 93.0% of the concentration measured in neat plasma overall (Table 11). Duplicate variation was similar in spiked and neat plasma specimens (Table 11).

**Table 11:** Duplicate variation and %concentration of neat plasma vs. plasma spiked with red blood cells

	Duplicate variation (%CV)			%concentration of neat plasma		
Red blood cell spike (% volume)	Sham animals (*n* = 3)	TBI animals (*n* = 3)	All animals (*n* = 6)	Sham animals (*n* = 3)	TBI animals (*n* = 3)	All animals (*n* = 6)
0%	8.2	1.1	4.6	–	–	–
	4.8	1.5	3.2	94.1	91.9	93.0
25%	9.6	1.4	5.5	77.1	67.6	72.4
	7.9	4.7	6.3	52.7	51.8	52.3

*CV* coefficient of variation

### Assay stability to modifications in blood collection and processing method

**Fig. 8:**
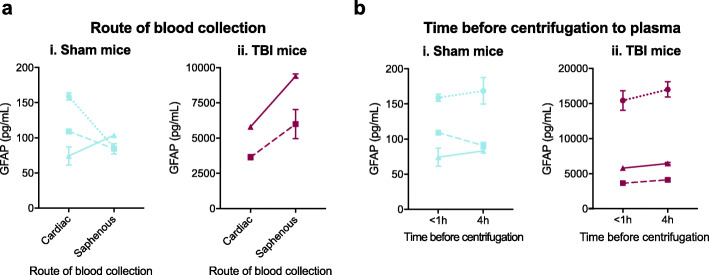
Assay measurements are unaffected by a delay in blood centrifugation to plasma but may be affected by route of blood collection. Blood was collected from mice 6 h after isoflurane exposure (sham) or isoflurane exposure and TBI by the saphenous vein and by cardiac puncture. Saphenous blood was immediately centrifuged to plasma. Blood collected by cardiac puncture was separated into two equal aliquots. One aliquot was immediately centrifuged to plasma (optimal) and one aliquot was left on ice for 4 h before centrifugation to plasma (4 h on ice). After centrifugation to plasma, samples were immediately stored at − 80 °C. Samples were assayed in duplicate. Mean plasma GFAP concentrations for each mouse are plotted with points representing mean and error bars ±SD of duplicates. The effect of blood collection and processing method on measured concentration was analyzed by paired *t* test; no significant differences were found. SD: standard deviation, TBI: traumatic brain injury

Analyte stability was also evaluated in response to modifications in methods of blood collection and processing. Blood was collected via cardiac puncture or saphenous vein bleed from 3 mice exposed to TBI and 3 sham mice 6 h after the TBI or sham procedure (Fig. 8a). In sham mice, plasma GFAP concentrations from blood collected by saphenous bleed were only 90.3 ± 44.3% of that collected by cardiac puncture (Fig. 8ai) (*p* = 0.5217 by paired *t* test) while in TBI mice, plasma GFAP concentrations from saphenous blood were 163.4 ± 1.8% of that from cardiac puncture (Fig. 8aii) (*p* = 0.1314 by paired *t* test) (Table 12). We also investigated the effect of delaying the centrifugation of blood intro plasma. Blood collected by cardiac puncture was aliquoted and one aliquot was centrifuged into plasma within 1 h while the other aliquot was incubated on ice for 4 h before centrifugation. Overall, delaying centrifugation of blood into plasma by 4 h had little effect on the concentrations of GFAP measured in plasma from sham (Fig. 8bi) or TBI (Fig. 8bii) animals compared to plasma centrifuged within 1 h of collection (*p* = 0.9919 and *p* = 0.1180 by paired *t* test for sham and TBI animals, respectively) (Table 12). Blood collection method and processing had little effect on duplicate variation as all duplicates were within 10% CV (Table 12).

**Table 12:** Impact of blood collection and processing method on assay performance

Sample handling	Mean duplicate CV (%)		%recovery* (mean ± SD) (***n*** = 3)	
	Sham mice	TBI mice	Sham mice	TBI mice
Cardiac puncture, centrifuged < 1 h after collection	7.8	4.7	–	–
Saphenous collection	5.1	9.4	90.3 ± 44.3	163.4 ± 1.8
4 h delay before centrifugation	5.4	3.3	100.5 ± 15.4	111.5 ± 1.5

*%recovery = (concentration measured with modified collection or processing/concentration measured with optimal collection and processing) × 100%. *CV* coefficient of variation, *SD* standard deviation

### Proof-of-concept analysis of plasma GFAP as a TBI blood biomarker in mice

**Fig. 9:**
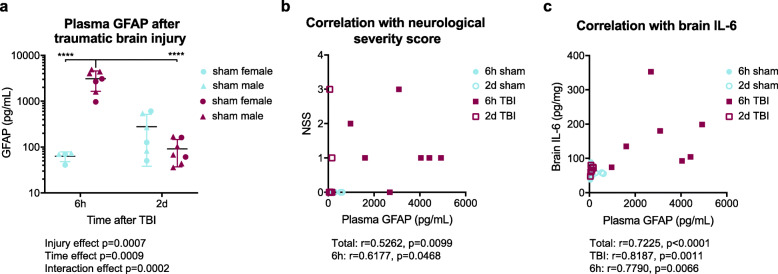
Murine plasma GFAP concentrations are acutely elevated after a closed-head TBI. Male and female C57Bl/6 mice aged 3.5–5 months were anesthetized with isoflurane then exposed to a 2.5 J closed-head TBI with a CHIMERA device or to isoflurane without TBI (sham). Blood was collected by cardiac puncture 6 h or 2 d after the sham or TBI procedure and centrifuged to plasma. **a** Mean plasma GFAP concentrations of each group are plotted with points representing individual mouse plasma samples and error bars representing ±SD. Circles represent female mice and triangles represent male mice. The effects of injury and time after injury on plasma GFAP concentrations were analyzed by two-way ANOVA (exact *p* values below graph) followed by Sidak’s multiple comparisons test (detailed within graph) where ****p < 0.0001. Correlations between plasma GFAP concentrations and **b** neurological severity score 2 h after sham or TBI procedure and **c** brain IL-6 concentrations were analyzed by Spearman correlation. Significant correlations are displayed below graphs with exact *p* values. TBI: traumatic brain injury, SD: standard deviation, IL-6: interleukin 6, CHIMERA: closed-head injury model of engineered rotational acceleration

In humans after TBI, blood GFAP levels are high in the first 24 h after TBI and decline to baseline or near baseline levels during recovery, with lengthened trajectories as injury severity increases [10, 11, 13, 24–26, 31]. To determine if comparable kinetics are found in a mouse TBI model, we collected plasma 6 h or 2 days after sham or TBI procedures with a CHIMERA device at an energy level that we previously determined to result in elevated plasma total tau and NF-L levels, increased brain cytokines levels, blood-brain barrier leakage, microstructural vascular abnormalities, and microgliosis [22]. Plasma GFAP was detectable with our novel assay in all mice and was significantly elevated in plasma collected 6 h after TBI compared to plasma collected 6 h after sham procedure (49-fold) or 2 days after TBI (31-fold) (Fig. 9a) (injury effect *p* = 0.0007, time effect *p* = 0.0009, interaction effect *p* = 0.0002 by two-way ANOVA; *p* < 0.0001 6 h post-TBI vs. 6 h post-sham and *p* < 0.0001 6 h post-TBI vs. 2 days post-TBI by Sidak’s multiple comparisons test). Furthermore, plasma GFAP concentrations were positively correlated with neurological impairment and neuroinflammation. Neurological severity score (NSS) was evaluated 2 h after injury and correlated with plasma GFAP concentrations across the entire cohort and specifically in samples collected 6 h after TBI or sham procedures (Fig. 9b) (Spearman *r* = 0.5262 and *p* = 0.0099 for entire cohort; Spearman *r* = 0.6177 and *p* = 0.0468 for 6 h samples). Significant correlations were also observed between plasma GFAP concentrations and brain IL-6 concentrations in the entire cohort, in plasma from TBI-exposed mice, and in plasma collected 6 h after TBI or sham procedures (Fig. 9c) (Spearman *r* = 0.7225 and *p* < 0.0001 for entire cohort; Spearman *r* = 0.8187 and *p* = 0.0011 for TBI samples; Spearman *r* = 0.7790 and *p* = 0.0066 for 6 h samples).

### Proof-of-concept analysis of plasma GFAP in APP/PS1 mice

**Fig. 10:**
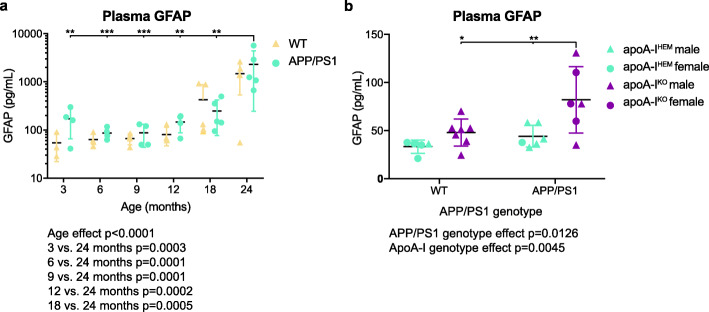
Plasma GFAP levels are elevated in aged APP/PS1 mice and APP/PS1 mice lacking apoA-I. **a** Female APP/PS1 mice (on a mixed C3H/Bl6 background) were aged to 3, 6, 9, 12, 18, or 24 months old then plasma specimens were collected by cardiac puncture. Mean plasma GFAP concentrations of each group are plotted with points representing individual mouse plasma samples and error bars representing ±SD. The effects of age and APP/PS1 genotype on plasma GFAP concentration were analyzed by two-way ANOVA (exact *p* values below graph) followed by Tukey’s multiple comparisons test across genotypes (exact *p* values below graph) or Sidak’s multiple comparison’s test within each genotype (detailed within graph, **p < 0.01, ***p < 0.001). **b** Male and female apoA-I hemizygous (apoA-I^HEM^) or knockout (apoA-I^KO^) mice with or without APP/PS1 transgenes (on a C57Bl/6 background) were aged to 12 months old then plasma specimens were collected by cardiac puncture. Mean plasma GFAP concentrations of each group are plotted with points representing individual mouse plasma samples and error bars representing ±SD. The effects APP/PS1 genotype and apoA-I genotype on plasma GFAP concentration were analyzed by two-way ANOVA (exact *p* values below graph) followed by Sidak’s multiple comparisons test (detailed within graph *p < 0.05, **p < 0.01). apoA-I: apolipoprotein A-I, SD: standard deviation

Recent studies have shown that serum or plasma GFAP may also be useful candidate blood biomarkers for AD. Serum GFAP was found to be elevated in AD subjects compared to controls in one study and plasma GFAP was found to predict amyloid positivity and associated with cognitive performance in another [20]. Given these findings, we measured plasma GFAP levels over the lifespan of APP/PS1 mice from 3 to 24 months of age (Fig. 10a). Overall, plasma GFAP was significantly affected by age (*p* < 0.0001 by two-way ANOVA) but not APP/PS1 genotype (*p* = 0.4873 by two-way ANOVA). Levels began to increase at 12 months of age and a significant increase was observed in 24-month-old mice compared to all other ages across genotypes (*p* = 0.0003, *p* = 0.0001, *p* = 0.0001, *p* = 0.0002, *p* = 0.0005 for 3-, 6-, 9-, 12-, and 18-month-old mice vs. 24-month-old mice, respectively, by Tukey’s multiple comparisons test) (Fig. 10a). Analysis of the aging effect within genotypes revealed that plasma GFAP concentrations were only significantly elevated in 24-month-old APP/PS1 mice and not in 24-month-old wildtype littermates (*p* = 0.0015, *p* = 0.0009, *p* = 0.0009, *p* = 0.0013, *p* = 0.0011 for 3, 6, 9, 12, and 18-month-old APP/PS1 mice vs. 24-month-old APP/PS1 mice, respectively, by Tukey’s multiple comparisons test) (Fig. 10a). Although APP/PS1 genotype had no significant effect on plasma GFAP levels, a trend was observed such that plasma GFAP levels were on average 1.6-fold higher in APP/PS1 mice compared to wildtype littermates across all age groups.

We next investigated whether apoA-I deficiency had any effect on plasma GFAP levels in APP/PS1 mice. Although AD is characterized by amyloid beta (Aβ) plaques and neurofibrillary tangles, cerebral vessel pathologies are also common [32, 33] and may be an early step in disease progression [34, 35]. Our group recently demonstrated that HDL, a class of circulating lipoproteins with known vasoprotective functions [36], may protect against cerebral vessel dysfunction in AD. We showed that plasma HDL may protect cerebral vessels from Aβ deposition and inflammation in 2D and 3D in vitro models of cerebral vessels [37–39]. We also previously studied the effects of HDL on cerebral vessels in vivo by crossing mice APP/PS1 mice with mice with loss-of-function mutations in apoA-I that lead to reduced plasma HDL-C levels [30]. We found that the lack of apoA-I in 12-month-old APP/PS1 mice exacerbated cortical amyloid deposition, cerebral vessel amyloid deposition, astrogliosis, and cerebral vessel-associated inflammation. Interestingly, we found that the cerebral vessels in APP/PS1 mice lacking apoA-I were associated with significantly more GFAP-positive astrocytes than cerebral vessels in APP/PS1 mice expressing apoA-I [30]. Given these previous observations of increased cerebral vessel pathology in apoA-I-deficient APP/PS1 mice, we investigated whether these mice also have elevated plasma GFAP levels. We observed a significant elevation in plasma GFAP levels in APP/PS1 mice compared to wildtype littermates overall (*p* = 0.0126 by two-way ANOVA) and in apoA-I-deficient mice compared to apoA-I-expressing mice overall (*p* = 0.0045 by two-way ANOVA) (Fig. 10b). Furthermore, mean plasma GFAP levels in APP/PS1 mice lacking apoA-I were 1.9-fold higher than APP/PS1 mice expressing apoA-I and 1.7-fold higher than WT mice lacking apoA-I (*p* = 0.0073 and *p* = 0.0119 by Sidak’s multiple comparisons test, respectively). Overall, these observations suggest that murine AD models develop increased plasma GFAP during aging and that GFAP may also be affected by circulating HDL levels, which have previously been shown to affect cerebral vessel health [30, 37–39].

## Discussion

Given the growing evidence for the utility of plasma or serum GFAP as a TBI biomarker with both diagnostic and prognostic contexts of use [4–13, 24–26, 31, 40, 41], we aimed to develop an immunoassay capable of measuring GFAP in mouse plasma to facilitate translational TBI studies using blood GFAP levels as an outcome. Rodent models remain critical tools in understanding biomechanical, histological, and biochemical outcomes after TBI, as they enable precise control over a wide variety of experimental injury conditions, have a clearly defined time of injury, and yield brain tissue specimens impossible to obtain in most studies of human TBI. Many assays of human GFAP have been developed with the Quanterix assay becoming an industry leader in the quantification of serum or plasma GFAP in humans [7, 12–14, 19, 42]. However, the Quanterix GFAP assay does not cross-react with murine GFAP [43]. Furthermore, commercial sandwich ELISAs for murine GFAP cannot reliably detect GFAP in plasma or serum and have a limited dynamic range (as described in Table 14). These are critical limitations as serum and plasma GFAP may be expected to have a large dynamic range in mouse models of TBI given the expected large dynamic range of serum and plasma GFAP observed in healthy humans and in humans after TBI [7–9, 24, 31, 44].

The novel GFAP immunoassay described here was developed for the Meso Scale Discovery platform, which uses ECL technology to provide high sensitivity, low background, and a broad dynamic range. We first obtained a commercial GFAP antibody pair from Abcam with established mouse, rat, and human cross-reactivity. The capture antibody was biotinylated to bind to streptavidin coated plates for increased sensitivity and the detection antibody was labeled with the MSD proprietary detection label. During plate reading, electrodes in the plate are activated resulting in a chemical reaction with bound detection label that produces light measured by the instrument [45].

We validated the analytical performance of our novel assay following the SOP suggested by Andreasson et al. [23] and compared its performance to that of GFAP assays for human blood and commercial ELISAs available for mouse GFAP. The most commonly used assays for measuring GFAP in human blood are the Banyan BTI assay and the Quanterix Neurology 4-plex assay. The Banyan assay was foundational for its use in studies leading to the FDA approval of GFAP, along with UCH-L1, as a biomarker for concussion [4]. In 2016, Quanterix licensed the GFAP antibody from the Banyan assay for use in their highly sensitive Simoa system [46], which is now the mostly commonly used assay for clinical studies [7, 12–14, 26, 31, 44]. Earlier GFAP biomarker studies used commercially available or prototype ELISAs from various companies [6, 11, 15, 16, 18, 41, 47].

Performance data for precision, limits of quantification, dilutional linearity, and recovery have been reported for some human and mouse assays and are summarized in Table 13 and Table 14. Data have not been routinely reported for robustness, parallelism, selectivity, and analyte stability. In terms of precision, the repeatability of our assay (5.0%) was very similar to that of the Banyan (3.6%) and Quanterix (4.2%) assays although the intermediate precision was considerably higher (7.2%) than the reported between-day CV of the human assays (0.8% and 0.0% for Banyan and Quanterix, respectively). Both precision parameters were very similar to those reported for commercial mouse ELISAs.

**Table 13:** Assay characteristics compared to human GFAP assays

Supplier		Quanterix^**#**^	Banyan^**#**^	BioVendor	Randox	Elecsys	Novel MSD assay
**Catalog number**		N4PA	BC-2209	RD192072200R	Stroke Array		
**Volume of sample/well (μL)**		152	250	35	N/A	50	12.5
**LLOD (pg/mL)**	**Outcome**	0.221	N/A	45	N/A	*50 †30	9
	**Protocol**	2.5 SD above background signal of blank. 2 reagent lots and 3 instruments, 12 runs total.	N/A	3 SD above background signal.	N/A	*2 SD above lowest standard point. †N/A	2.5 SD above background signal of 16 blank replicates.
**LLOQ (pg/mL)**	**Outcome**	0.467	10	N/A	160	N/A	< 24.8
	**Protocol**	Serial dilutions of calibrator assayed in triplicate. 2 reagent lots and 3 instruments, 12 runs total.	Lowest measured concentration of serum pools (*n* = 7, some spiked) with duplicate CV < 15%.	N/A	N/A	N/A	10 SD above background signal of 16 blank replicates. Lowest measured concentration of plasma specimens (*n* = 8) with duplicate CV < 20%.
**ULOQ (pg/mL)**	**Outcome**	4000	320	25,000	100,000	*150,000 †100,000	> 16,534
	**Protocol**	N/A	Highest measured concentration of serum pools (*n* = 7, some spiked) with duplicate CV < 15%.	N/A	N/A	N/A	Highest measured concentration of plasma specimens (*n* = 8) with duplicate CV < 20%.
**Intra-assay CV (%)**	**Outcome**	4.2	3.6	5.1	3–4	*1.1–1.9 †0.8–0.62	5.0
	**Protocol**	Within-run CV from 5 days, 2 runs/day, 1 plasma pool/run, 3 replicates/pool.	Within-run CV from 5 days, 1 run/day, 5 serum pools/run, 4 replicates/pool.	Within-run CV from 8 replicates of 2 serum specimens.	N/A	*Within-run CV from 1 run, 4 serum specimens, 21 replicates/specimen. †Within-run CV from 1 run, 5 serum specimens, 21 replicates/specimen.	Within-run CV from 5 days, 1 run/day, 3 plasma pools/run, 5 replicates/pool.
**Inter-assay CV (%)**	**Outcome**	0.0	0.8	5.7	N/A	*2.7–4.2 †1.8–5.9	7.2
	**Protocol**	Between-day CV from 5 days, 2 runs/day, 1 plasma pool/run, 3 replicates/pool.	Between-day CV from 5 days, 1 run/day, 5 serum pools/run, 4 replicates/pool.	Between-run CV from 7 runs, 2 serum specimens/run.	N/A	*Between-day CV from 10 runs, 4 serum specimens/run, 21 replicates/specimen †Between-day CV from 10 runs, maximum 2 runs/day, 5 serum specimens/run, 6 replicates/specimen.	Between-day CV from 5 days, 1 run/day, 3 plasma pools/run, 5 replicates/pool
**Recovery**	**Outcome**	50–120%	N/A	83–115%	N/A	N/A	62–97%^A^ 86–105%^B^
	**Protocol**	Spike 80 and 800 pg/mL into serum (*n* = 2) and plasma (*n* = 2).	N/A	Spike 500, 1000, and 5000 pg/mL into serum (*n* = 3).	N/A	N/A	Spike 100, 1000, and 10,000 pg/mL into plasma (*n* = 5).
**Dilution Linearity**	**Outcome**	102–121%	89–100%	97–121%	N/A	N/A	93–105%
	**Protocol**	Spiked serum diluted 1:4 to 1:128.	Combined clinical specimens with a low-concentration serum pool in different proportions (*n* = 11)	Serum (*n* = 3) diluted 1:2 to 1:8.	N/A	N/A	Plasma (*n* = 3) diluted 1:2 to 1:16.
**Performance data retrieved from:**		Supplier	Supplier	Supplier	Posti et al 2017 [47]	*Tichy et al [15] †Luger et al [18]	
**References**		[7, 11–14, 26, 31, 44]	[4, 8, 9, 17, 24, 25, 40]	[6, 10, 11]	[16, 41, 47]	[15, 18]	

^#^Same antibody pair used between assays. ^A^ Recovery data for samples with low endogenous GFAP. ^B^ Recovery data for samples with high endogenous GFAP. *N/A* data not available, *LLOD* lower limit of detection, *LLOQ* lower limit of quantification, *ULOQ* upper limit of quantification, *CV* coefficient of variation, *SD* standard deviation

**Table 14:** Assay characteristics compared to existing commercial ELISA assays for mice

Supplier		MyBioSource					Abcam^*^	ELISA Genie	Millipore	Cusabio	Novel MSD assay
**Catalog number**		MBS723651	MBS2515511	MBS2018967	MBS763852	MBS2701012	ab233621	MOFI00191	NS830	E08603m	
**Cost/plate**		$640 USD	$490 USD	$625 USD	$415 USD	$350 USD	$593 CAD	€599	$693 CAD	$600 USD	$150 USD
**Volume of sample (μL)**		50	100	100	100	100	50	100	100	50–100	12.5
**Detection range (pg/mL)**		50–1000	15.63–1000	15.6–1000	15.6–1000	62.5–4000	125–8000	15.6–1000	1500–100,000	3.12–200	24.8–16,533.9
**LLOD (pg/mL)**	**Outcome**	1	9.38	< 6.3	9.38	23.3	8.7	9.375	1500	0.78	9
	**Protocol**	N/A	N/A	2 SD above background of 20 blanks replicates.	N/A	2 SD above background of 20 blank replicates.	N/A	N/A	N/A		2.5 SD above background 16 blank replicates
**Intra-assay CV (%)**	**Outcome**	< 9	4.6	< 10	< 8	< 10	4.4	< 8	6.8	< 8	5.0
	**Protocol**	N/A	Within-run CV from 1 run, 3 specimens, 20 replicates/specimen.	Within-run CV from 1 run, 3 specimens, 20 replicates/specimen.	Within-run CV from 1 run, 3 specimens, 20 replicates/specimen.	Within-run CV from 1 run, 3 specimens, 20 replicates/specimen.	Within-run CV from 1 run, 8 replicates.	Within-run CV from 1 run, 3 specimens, 20 replicates/specimen.	Within-run CV from 1 run, 3 specimens, 16 replicates/specimen.	Within-run CV from 1 run, 3 specimens, 20 replicates/specimen.	Within-day CV from 5 days, 1 run/day, 3 plasma pools/run, 5 replicates/pool.
**Inter-assay CV (%)**	**Outcome**	< 10	5.1	< 12	< 10	< 12	5.8	< 10	5.2	< 10	7.2
	**Protocol**	N/A	Between-run CV from 3 runs, 3 specimens/run, 20 replicates/specimen.	Between-run CV from 3 runs, 3 specimens/run, 8 replicates/specimen.	Between-run CV from 3 runs, 3 specimens/run, 8 replicates/specimen.	Between-run CV from 3 runs, 3 specimens/run, 8 replicates/specimen.	Between-run CV from 3 runs.	Between-run CV from 3 runs, 3 specimens/run, 8 replicates/specimen.	Between-run CV from 3 runs, 6 specimens/run, 2 replicates/specimen.	Between-run CV from 20 runs, 3 specimens/run.	Between-day CV from 5 runs, 3 plasma pools/run, 5 replicates/pool.
**Recovery**	**Outcome**	94–103%	89–103%	78–99%	85–105%	80–98%	95–100%	88–105%	95–109%	90–104%	62–97%^A^ 86–105%^B^
	**Protocol**	N/A	Spiked serum, plasma, culture media (*n* = 5 each) at 3 concentrations.	Spiked plasma (*n* = 10) and serum (*n* = 5).	Spiked plasma (*n* = 10) and serum (*n* = 5).	Spiked plasma (*n* = 10) and serum (*n* = 5).	Spiked brain extract.	Spiked plasma (*n* = 10) and serum (*n* = 5).	Spike brain (*n* = 1) or cell (*n* = 2) lysate with 1.56–50 ng GFAP.	Spiked serum (*n* = 5) or plasma (*n* = 5).	Spike plasma (*n* = 5) with 100, 1000, and 10,000 pg/mL GFAP.
**Dilution linearity**	**Outcome**	92–108%	80–109%	80–108%	81–104%	78–105%	87–100%	81–104%	77–122%	85–108%	93–105%
	**Protocol**	2× to 16×	Spiked serum, plasma, culture media (*n* = 5 each), diluted 1:2 to 1:16.	Spiked plasma (*n* = 10) and serum (*n* = 5), diluted 1:2 to 1:16.	Spiked plasma (*n* = 10) and serum (*n* = 5), diluted 1:2 to 1:16.	Spiked plasma (*n =* 10) and serum (*n =* 5), diluted 1:2 to 1:16.	Brain extract (*n =* 2) diluted 1:2 to 1:16.	Spiked plasma (*n =* 10) and serum (*n =* 5), diluted 1:2 to 1:16.	Spiked brain lysate (*n =* 3) 1:3 to 1:27.	Spiked serum (*n* = 4), diluted 1:1 to 1:8.	Plasma (*n =* 3) diluted 1:2 to 1:16.

^*^Same antibody pair used between assays. ^A^ Recovery data for samples with low endogenous GFAP. ^B^ Recovery data for samples with high endogenous GFAP. *LLOD* lower limit of detection, *CV* coefficient of variation, *SD* standard deviation. Data retrieved from supplier product websites

The limits of quantification of our assay have some advantages and some disadvantages compared to other assays. Although our assay is less sensitive than the Quanterix assay and some commercial mouse ELISAs, it has a large detection range and a similar LLOQ to the Banyan assay. Importantly, all plasma specimens assayed in this study, including those from uninjured wildtype mice, were detectable by our assay with the lowest concentration measured at 24.8 pg/mL in plasma from a female 2.5-month-old C57Bl/6 mouse, well above the calculated LLOD of 9 pg/mL.

We assessed the dilution linearity of plasma specimens with high endogenous GFAP concentration up to a 64-fold dilution. Dilution linearity up to a 16-fold dilution was similar or better than that reported for the Quanterix and Banyan assays, although the Quanterix assay showed better linearity over a larger dilution range of up to 132-fold. By this metric, our assay also performed better than the commercial mouse ELISAs. However, linearity of the commercial mouse ELISAs was mainly established by spiking plasma, serum, or media samples with recombinant GFAP and diluting the spiked samples. Using this approach, the dilution linearity of our assay was far superior to the commercial ELISAs with 91–123% linearity up to a 100,000-fold dilution.

To assess recovery, we spiked specimens with low and high endogenous GFAP with recombinant GFAP at 3 different concentrations. Mean spike recovery of our assay was superior to that reported for the Quanterix assay. Spike recovery performance has not been reported for the Banyan assay. Although spike recovery for low-concentration specimens was poor compared to data from commercial mouse ELISAs, spike recovery for high concentration specimens was comparable.

Having validated important analytical parameters of this novel assay, we next examined the ability of our assay to detect GFAP in mouse plasma with kinetics that resemble that of the human GFAP response after TBI. We used the non-surgical CHIMERA model of TBI for this proof-of-concept experiment. Plasma GFAP was detectable in all injured and uninjured specimens and we observed an acute, robust increase in plasma GFAP after TBI. Specifically, GFAP levels were 49-fold higher in plasma collected 6 h after TBI compared to plasma collected 6 h after the sham procedure. By 2 days after injury, GFAP levels did not differ significantly between TBI and sham mice. This acute response of plasma GFAP to TBI resembles the acute response of GFAP in studies of human TBI where GFAP levels are found to peak within 24 h of injury [11, 24–26]. Longer follow-up studies have suggested that the long-term response of GFAP may be biphasic with an initial reduction after injury followed by an increase after 6 months [31]. Additional future studies will be needed to determine the precise temporal profiles of plasma GFAP after TBI or other neurological insults in mice both at acute and chronic timepoints.

We also performed preliminary analyses of the associations between plasma GFAP and TBI outcomes in mice. Plasma GFAP correlated with neurological severity scores evaluated 2 h after the TBI or sham procedure across the entire cohort and specifically in plasma specimens collected 6 h after sham or TBI. As previously discussed, blood GFAP levels have been shown to associate with several outcomes after TBI in many human studies. For example, serum GFAP levels were higher after concussion in subjects experiencing loss of consciousness or posttraumatic amnesia than in concussion subjects without these conditions [26]. In CT-positive subjects, subjects with acute unfavorable outcomes, including death or craniotomy, had increased serum GFAP compared to CT-positive subjects with stable, improving, or transient neurological dysfunction [9]. Blood GFAP also associates with injury severity measured with Glasgow Comac Scale [10, 40, 41] although associations may not exist when investigating exclusively mild TBI [6]. Many TBI outcomes in human studies are evaluated 6 to 12 months after injury [5, 6, 11, 13]; therefore, future studies will need to be performed to determine whether blood GFAP levels also predict long-term outcomes after TBI in mice.

TBI is only one of many neurological indications for which blood biomarkers may be useful. Advances in assay technology have also aided the development of blood biomarkers for AD including Aβ42/40, phosphorylated tau, and neurofilament light [48]. GFAP has thus far been less studied as a blood biomarker for AD but recent investigations have found that it is elevated in AD subjects [19], can predict brain amyloid positivity [20], and associates with cognitive function [20]. Therefore, we aimed to provide preliminary data on GFAP in the blood of AD model mice using our novel assay. In APP/PS1 mice, a common AD model, we observed an age-dependent increase in plasma GFAP starting at 12 months of age, around the time at which amyloid plaques are well-established [49]. By 24 months of age, plasma GFAP levels were significantly higher than levels at 3, 6, 9, 12, or 18 months of age. Although plasma GFAP concentrations tended to be higher in APP/PS1 mice compared to wildtype littermates at most ages, the difference was not significant.

Intriguingly, we observe elevated plasma GFAP levels in 12-month-old APP/PS1 mice with apoA-I-deficiency. ApoA-I is the primary protein component of plasma HDL therefore apoA-I-deficient mice have drastically reduced plasma HDL-C levels [50]. We previously showed that plasma HDL has vasoprotective functions on in vitro models of cerebral vessels [37–39] and apoA-I deficiency exacerbates cortical amyloidosis, astrogliosis, and vascular inflammation in APP/PS1 mice [30]. Mice have relatively high vascular resilience compared to humans due to their high plasma HDL to low-density lipoprotein ratios [51] and lack of exposure to modifiable risk factors, which account for up to 40% of dementia risk in humans [52]. Therefore, our data suggest that although amyloidosis on its own may not cause elevated plasma GFAP in mice, combining amyloidosis with factors that reduce vascular resilience, such as apoA-I deficiency, may better recapitulate changes in plasma GFAP observed in human AD subjects.

Notably, our study compares apoA-I-deficient mice to mice with apoA-I haploinsufficiency rather than mice with wildtype apoA-I expression levels due to survival issues with apoA-I-deficient mice that have previously been thoroughly explained [30]. Previous studies have found a greater magnitude difference in plasma apoA-I levels between mice with total apoA-I deficiency and apoA-I haploinsufficiency compared to the difference between mice with wildtype apoA-I levels and mice with haploinsufficiency [50]. Furthermore, we have previously shown a robust reduction in plasma HDL-C levels in apoA-I-deficient mice compared to mice with apoA-I haploinsufficiency [30].

The ability to complement the many exceptional ongoing clinical biomarker studies with preclinical data from mouse models is a considerable advance for the field. Studying TBI exclusively in humans is challenged by considerable heterogeneity in TBI severity, mechanism of injury, age at injury, and many other variables. Although the majority of TBI cases are classified as mild, many of these cases may not seek medical attention or be admitted to hospital for treatment or enrollment into a clinical research study [53]. TBI studies in animal models allow for a level of control over injury severity and sample collection not possible in human studies. Studying TBI in mouse models also allows for more extensive analysis of how blood biomarkers relate to changes in brain tissue through histological and biochemical analyses. We began to make use of this advantage in the current study in our analysis of brain cytokine concentrations 6 h and 2 days after sham or TBI procedures. We found that plasma GFAP concentrations correlated with brain IL-6 concentrations in mice exposed to TBI and sham injuries. Mouse models also have important roles in preclinical drug testing and for experimental investigation of the interactions of TBI with co-morbid conditions, such as Alzheimer’s disease [54, 55] and anabolic steroid use [56]. We are encouraged by the potential ability to measure TBI blood biomarkers in serial mouse plasma specimens, as this may catalyze profound advancements and reduce animal numbers needed for a preclinical study by shifting to longitudinal analyses that include within-subject outcomes.

Although GFAP has shown great promise as a blood biomarker for TBI, some limitations remain. A 2017 study by Posti et al. measured serum GFAP in people with orthopedic injuries without CNS involvement and found levels higher than or the same as levels in CT-negative mild TBI patients [47]. The authors therefore urged caution in the use of GFAP in diagnosing mild TBI in orthopedic trauma patients. The use of GFAP as a diagnostic biomarker may also be hindered by the relatively narrow time window after injury during which it is elevated. Studies have consistently shown that blood GFAP levels peak within the first 24 h after injury and then decline [11, 24–26]. Future studies will need to be performed to determine whether the long-term temporal profile of blood GFAP after TBI in mice reflects that of humans and the specificity to CNS injury. One potential limitation in performing these studies is the need for interim blood collection. We performed preliminary tests comparing GFAP levels in blood collected by cardiac puncture compared to blood collected from the saphenous vein in the same mouse in order to address this question. Although only 12.5 μL of plasma is required to measure GFAP in our novel assay, technical difficulties in obtaining blood via the saphenous vein may still limit serial GFAP measurement, as occurred in our preliminary study presented here. Our preliminary study also suggested a potential bias in the measured concentration based on blood collection method. No significant differences were found between blood collection or processing methods by paired *t* tests; however, variations in the measurements were substantial and further studies assessing these pre-analytical factors will be important. GFAP concentration in blood collected via the saphenous vein of TBI-exposed mice was on average 163% of that measured in blood collected via cardiac puncture from the same animal. As blood was collected from the saphenous vein on average 92 min before collection via cardiac puncture, it is possible that the differences in GFAP concentrations could be due to the rapid changes in circulating GFAP levels that occur in the first few hours after TBI [24, 25]. Differences may also be the result of differences in the materials and techniques used to collect blood via cardiac puncture vs. saphenous vein. An additional concern with respect to saphenous vein blood collection is the increased risk of specimen hemolysis as we found that hemolysis can suppress measured GFAP concentrations in our assay.

### Limitations

This study has several limitations. First, this study must be considered a partial validation. Although this study evaluated most of the immunoassay validation parameters proposed by Andreasson et al., we did not evaluate trueness or uncertainty and performed only a limited evaluation of selectivity [23]. Protocols for the validation of assay trueness and uncertainty provided by Andreasson et al. require comparison to a certified reference material [23]. As certified reference materials for mouse plasma GFAP are not available, validation of these parameters was not possible. However, if this assay is to be applied to human specimens in the future, validation of assay trueness and uncertainty should be considered. Our analysis of selectivity was limited to investigating serum and plasma specimens and to investigating the effects of specimen hemolysis. Future studies will further evaluate selectivity by testing the ability of the assay to detect GFAP in additional matrices and in the presence of confounding factors. Furthermore, validation protocols were only performed in a single laboratory; therefore, future cross-laboratory validation experiments will be necessary to fully understand the performance of this assay. The validation of the assay was also limited in that only a single monoclonal antibody pair was tested. The antibody pair used was selected for its advertised species reactivity, the buffer in which the antibodies were dissolved, and the relatively large quantities available for purchase. However, alternative antibody pairs could be investigated in the future and may also be effective in this assay.

In addition to limitations with respect to assay validation parameters, this study is also limited in the types of specimens tested. Future studies will be needed to assess whether this assay can detect GFAP in fluids or tissues from other species. Studies to assess the ability of this assay to detect GFAP in human, rat, pig, and ferret blood specimens are ongoing and preliminary experiments show promise for the utility of this assay in these species. Finally, although we described how our assay performs compared to existing human and mouse GFAP in Table 13 and Table 14, we have not directly compared any of these assays to our assay on the same samples in our laboratory.

## Conclusions

We developed the first known immunoassay capable of quantifying GFAP in murine plasma. Having established its analytical rigor, we used this assay to reveal a murine plasma GFAP response to CHIMERA TBI that is similar to the GFAP response to TBI in humans. We also provide preliminary data using this assay to study plasma GFAP in AD model mice. Future studies with this assay have the potential to bridge the gap between clinical and preclinical TBI research and accelerate the understanding of TBI pathophysiology and development of TBI therapeutics.

## Supplementary Information

**Additional file 1.** GFAP assay precision mixed model output. Exported SPSS Statistics output analyzing precision data (“Repeatability and intermediate precision of the assay” section, Fig. 2, Table 5). (XLSM 57 kb)

## Data Availability

The data sets used and analyzed during the current study are available from the corresponding author on reasonable request.

## Abbreviations

GFAP: Glial fibrillary acidic protein
TBI: Traumatic brain injury
CT: Computed tomography
MRI: Magnetic resonance imaging
CHIMERA: Closed-head impact model of engineered rotational acceleration
AD: Alzheimer’s disease
CSF: Cerebrospinal fluid
UCH-L1: Ubiquitin carboxy-terminal hydrolase L1
FDA: Food and Drug Administration
ELISA: Enzyme-linked immunosorbent assay
MS: Multiple sclerosis
HDL: High-density lipoprotein
RT: Room Temperature
MSD: Meso Scale Discovery
EDTA: Ethylenediaminetetraacetic acid
PBS: Phosphate-buffered saline
BIOMARKAPD: Biomarkers for Alzheimer’s disease and Parkinson’s disease
SOP: Standard operating procedure
rpm: Revolutions per minute
CV: Coefficient of variation
SD: Standard deviation
ECL: Electrochemiluminescence
LLOD: Lower limit of detection
RIPA: Radioimmunoprecipitation
BCA: Bicinchoninic acid
LLOQ: Lower limit of quantification
ULOQ: Upper limit of quantification
HDL-C: High-density lipoprotein cholesterol
apoA-I: Apolipoprotein A-I
